# Supplementary material for: Limits to Electrical Mobility in Lead-Halide Perovskite Semiconductors
Source: J Phys Chem Lett. 2021 Apr 6;12(14):3607–17. doi: 10.1021/acs.jpclett.1c00619 (PMC8154852; doi:10.1021/acs.jpclett.1c00619)
Supplement: Supplementary file 1 — jz1c00619_si_001.pdf [file jz1c00619_si_001.pdf]

Supporting Information: Limits to Electrical Mobility in Lead-Halide Perovskite Semiconductors

Chelsea Q. Xia,[†] Jiali Peng,[‡] Samuel Poncé,[¶,§] Jay B. Patel,[†] Adam D. Wright,[†] Timothy W. Crothers,[†] Mathias Uller Rothmann,[†] Juliane Borchert,[†] Rebecca L. Milot,[‖] Hans Kraus,[⊥] Qianqian Lin,[‡] Feliciano Giustino,[¶,#,@] Laura M. Herz,[†] and Michael B. Johnston[*,†]

†*Department of Physics, University of Oxford, Clarendon Laboratory, Parks Road, Oxford OX1 3PU, U.K.*

‡*Key Lab of Artificial Micro- and Nano-Structures of Ministry of Education of China, School of Physics and Technology, Wuhan University, Wuhan 430072, P. R. China*

¶*Department of Materials, University of Oxford, Parks Road, Oxford OX1 3PH, U.K.*

§*Theory and Simulation of Materials (THEOS), École Polytechnique Fédérale de Lausanne, CH-1015 Lausanne, Switzerland*

‖*Department of Physics, University of Warwick, Gibbet Hill Road, Coventry CV4 7AL, U.K.*

⊥*Department of Physics, University of Oxford, Denys Wilkinson Building, Keble Road, Oxford OX1 3RH, U.K.*

#*Oden Institute for Computational Engineering and Sciences, University of Texas at Austin, Austin, Texas 78712, USA*

@*Department of Physics, University of Texas at Austin, Austin, Texas 78712, USA*

E-mail: michael.johnston@physics.ox.ac.uk

Contents

1 Terahertz spectroscopy setup **S-3**

2 Derivation of charge-carrier electrical mobility **S-5**

2.1 Mobility measured in transmission S-5

2.2 Mobility measured in reflection S-9

2.3 Literature values of electrical mobility in $MAPbI_3$ S-14

3 Effect of grain-boundary scattering on electrical mobility **S-17**

3.1 Theoretical modeling S-17

3.2 Grain-size measurement of polycrystalline $MAPbI_3$ thin film S-20

4 Significance of photon-reabsorption effects **S-22**

4.1 Charge-carrier dynamics analysis S-22

4.2 Charge-carrier diffusion length calculation S-27

4.3 Photoconductivity decay dynamics of thin film measured in reflection S-31

5 PL-facilitated temperature correction of $MAPbI_3$ single crystal **S-32**

1 Terahertz spectroscopy setup

Figure S1 shows the experimental setup for the optical pump terahertz probe spectroscopy (OPTPS) in both transmission and reflection modes. An amplified laser (with central wavelength of 800 nm, 5 kHz repetition rate, 35 fs pulse width and 4 W average power) was split into three arms: gate beam, THz beam and pump beam. The THz pulse was generated by a spintronic emitter due to inverse spin Hall effect.[S1] The pump beam was up-converted to 400 nm by a β-barium-borate (BBO) crystal and a short-pass filter was used to filter out the residual 800 nm light. Two optical choppers were used to reduce the repetition rates of the THz and pump beam to 2.5 kHz and 1.25 kHz, respectively. The resultant THz signal was detected by electro-optic sampling using a set of a (110) ZnTe crystal, a quarter-wave plate, a Wollaston prism and a pair of balanced photodiodes. A silicon beamsplitter was used to direct the reflected THz signal from the sample to another detection arm. The photoluminescence (PL) spectrum of the sample was induced by the 400 nm pump beam and collected by a fiber-coupled CCD spectrometer (Horiba iHR320). The sample was mounted on a cold-finger cryostat which was cooled down by liquid helium and able to vary the sample temperature from 5 K up to 370 K.

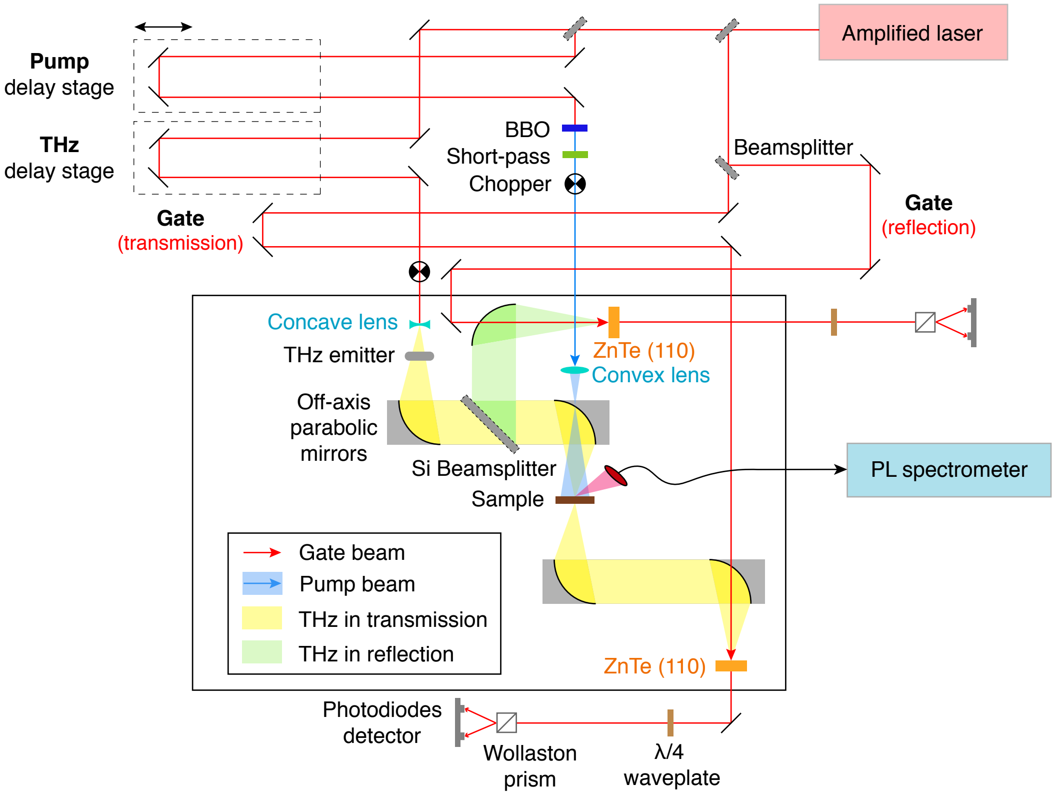

Figure S1: Schematic of the experimental setup for OPTPS. An amplified laser pulse is split into three beam paths: gate beam, pump beam and THz beam. The THz signal is generated by a spintronic emitter and detected by a ZnTe crystal via electro-optic sampling. The PL spectrum is collected by a fiber-coupled CCD spectrometer.

2 Derivation of charge-carrier electrical mobility

2.1 Mobility measured in transmission

When measuring the electrical mobility in the transmission mode of OPTPS, the ratio between the change of THz signal and transmitted THz signal, $\Delta T/T$, is defined as

$$\frac{\Delta T}{T} \equiv \frac{E_{\text{on}} - E_{\text{off}}}{E_{\text{off}}} = \frac{\Delta E_t}{E_t}, \tag{S1}$$

where the subscripts "on" and "off" mean photoexcitation and non-photoexcitation, respectively. The photo-induced field change, $\Delta E(z)$, satisfies the wave equation given below,

$$\frac{d^2 \Delta E(z)}{dz^2} + k^2 \Delta E(z) = -ik_0 Z_0 \Delta\sigma(z) E(z), \tag{S2}$$

where $k_0 = \omega/c$ is the wave vector of the THz field in vacuum and $k = nk_0$ is the wave vector inside the sample, where n is the refractive index of the sample in THz frequency range. In the case of $MAPbI_3$, $n = \sqrt{\varepsilon_{\text{stat}}} = \sqrt{30}$ which is an average of previously reported values measured in the THz-GHz frequency range.[S2–S4] $Z_0 = \varepsilon_0^{-1} c^{-1}$ is the vacuum wave impedance and $\Delta\sigma(z)$ is the photoconductivity of the sample which varies with the depth z following the Beer-Lambert law,

$$\Delta\sigma(z) = \Delta\sigma_0 \exp(-i\alpha z), \tag{S3}$$

where $\Delta\sigma_0 = \Delta\sigma(0)$ is the photoconductivity at the front surface of the sample and α is the absorption coefficient of the sample at the photoexcitation wave length 400 nm, which can be measured by a Fourier-transform infrared (FTIR) spectrometer. Conventionally, the photoconductivity can be expressed in terms of $\Delta T/T$ as[S5]

$$\Delta\sigma(\omega) = -\frac{\varepsilon_0 c(1+\tilde{n}_{\text{s}})}{L} \left(\frac{\Delta T}{T}\right), \tag{S4}$$

where $\tilde{n}_s$ is the complex refractive index of the substrate, which in our experiment is the z-cut quartz disc. $L = 1/\alpha$ is the thickness of the photoexcited layer of $MAPbI_3$ thin film. Equation (S4) can be used for converting the experimentally measured $-\Delta T/T$ to photoconductivity $\Delta\sigma$ as shown in Figure 2a in the main text. Combining Equations (S2) and (S3), the general solution can be expressed in terms of the electrical mobility μ as[S6]

$$\mu = \frac{n_1 + n_2}{2n} \frac{a}{e\Phi_{\text{eff}}} \left[(1 + r_1 r_2 \exp(2ikL)) \int_0^L \Delta\sigma(z) dz \right.$$
$$\left. + r_1 \int_0^L \exp(2ikz) \Delta\sigma(z) dz + r_2 \exp(2ikL) \int_0^L \exp(-2ikz) \Delta\sigma(z) dz \right], \quad \text{(S5)}$$

where n_1 and n_2 are refractive indices of the medium surrounding the sample, which in the case of the $MAPbI_3$ thin film are air and quartz, respectively. $r_1 = (n - n_1)/(n + n_1)$ and $r_2 = (n - n_2)/(n + n_2)$ are the reflection coefficients at the front and back surfaces of the thin film respectively, and $a = [1 - r_1 r_2 \exp(2ikL)]^{-1}$ describes the multiple internal reflections due to Fabry-Perot effect. The effective photon fluence Φ_{eff} is defined as

$$\Phi_{\text{eff}} = \frac{N}{A_{\text{eff}}}, \quad \text{(S6)}$$

where N is the number of charge carriers induced by the pump beam,

$$N = \varphi \frac{E\lambda}{hc} (1 - R_{\text{pump}})(1 - T_{\text{pump}}), \quad \text{(S7)}$$

with E being the energy of pump pulse at wavelength $\lambda = 400\,\text{nm}$. R_{pump} and T_{pump} are the reflectivity and transmissivity of the sample at 400 nm respectively, which can be measured by FTIR spectroscopy. $\varphi \leq 1$ is the ratio of free charge-carriers created per photon absorbed, which in the case of $MAPbI_3$ is assumed to be unity.

Distinct from the pump photon fluence (which is a mere measurement of the pump beam), the effective photon fluence, Φ_{eff}, while taking into account the conversion ratio between absorbed photons and induced charge carriers, is also related to the photoconductive part

of the sample, which is determined by the overlapping area between the pump beam and the THz beam. In other words, Φ_{eff} is essentially the photo-induced charge-carrier density weighted by the THz electric field strength at each pump beam spatial position. Therefore, to calculate this effective "absorption intensity", we need to use the following weighting function,

$$\frac{N}{A_{\text{eff}}} = \int_0^{2\pi}\int_0^{\infty} \hat{n}_{\text{pump}} g_{\text{THz}} r dr d\theta, \tag{S8}$$

where $\hat{n}_{\text{pump}}$ is the two-dimensional (2D) areal charge-carrier density induced by the pump beam, which is proportional to its photon density by a factor of φ and g_{THz} is the normalised THz areal spread function. We assume that $\hat{n}_{\text{pump}}$ and g_{THz} follow 2D Gaussian distribution,

$$\hat{n}_{\text{pump}}(r) = \frac{N}{2\pi\sigma_{\text{pump}}^2} \exp\left(-\frac{r^2}{2\sigma_{\text{pump}}^2}\right), \tag{S9}$$

$$g_{\text{THz}}(r) = \frac{1}{2\pi\sigma_{\text{THz}}^2} \exp\left(-\frac{r^2}{2\sigma_{\text{THz}}^2}\right), \tag{S10}$$

where σ_{pump} and σ_{THz} are the beam waists of the pump and THz pulses respectively. r is the radial distance from the center of the Gaussian distribution. It can be shown that $\int_0^{2\pi}\int_0^{\infty} \hat{n}_{\text{pump}} r dr d\theta = N$ and $\int_0^{2\pi}\int_0^{\infty} g_{\text{THz}} r dr d\theta = 1$. We represent $\hat{n}_{\text{pump}}$ and g_{THz} as blue and red curves respectively in Figure S2, where their beam waists, σ_i, are related to their full-width-at-half-maximum (FWHM) by,

$$\sigma_i = \frac{\text{FWHM}_i}{2\sqrt{2\ln 2}}. \tag{S11}$$

Combining Equations (S8)–(S10), we obtain the expression for the effective area,

$$A_{\text{eff}} = 2\pi(\sigma_{\text{pump}}^2 + \sigma_{\text{THz}}^2). \tag{S12}$$

According to the thin-film approximation (TFA), in which the thickness of the photoexcited layer of the sample, $L = 1/\alpha$, is much smaller than the smallest wavelength in the

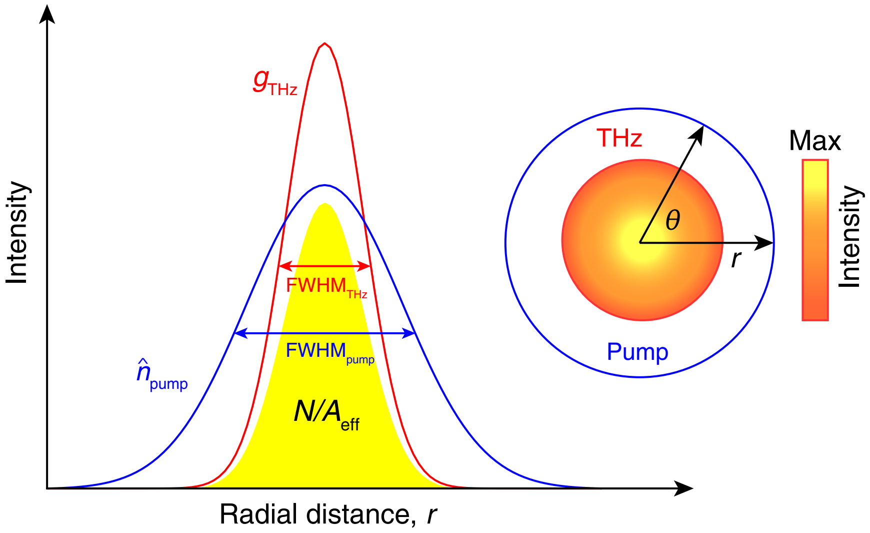

Figure S2: Gaussian profiles of the pump-induced charge-carrier density and normalised THz intensity distribution. The yellow area represents the effective charge-carrier density within the overlapping area between the pump and THz beams. The inset is a 2D illustration of the overlapping between the pump and THz beams.

THz pulse,[S7] we assume the unexcited part of the $MAPbI_3$ thin film to be the same as the substrate. Therefore, assuming $n_0 \approx n_2$ and $r_2 \approx 0$, Equation (S5) can be simplified as

$$\mu = \frac{\Delta S A_{\rm eff}}{Ne}, \tag{S13}$$

where $\Delta S = \int \Delta\sigma(z)dz$ is the sheet conductivity that can be expressed in terms of the measured changed of THz signal $\Delta T/T$,

$$\Delta S = -\varepsilon_0 c(n_1 + n_2)\left(\frac{\Delta T}{T}\right), \tag{S14}$$

which leads to our final expression for the effective mobility in transmission,

$$\varphi\mu_{\rm T} = -\varepsilon_0 c(n_1 + n_2)\frac{A_{\rm eff}hc}{Ee\lambda(1 - R_{\rm pump})(1 - T_{\rm pump})}\left(\frac{\Delta T_{\rm pp}}{T_{\rm pp}}\right). \tag{S15}$$

The photoconductivity term $\Delta T_{\rm pp}/T_{\rm pp}$ is measured at the instant of photoexcitation, $t = 0$, which is at the peak of the photoconductivity decay curve shown in Figure 2 in the

main text. The change in charge-carrier density due to carrier recombination, diffusion and photon reabsorption, which happens at later times, is therefore not relevant in the calculation of charge-carrier mobility. It is also worth noting that the mobility expression given by Equation (S15) is slightly different from the conventional one which uses the value of $\Delta T/T$ at the peak of THz pulse (i.e. $\Delta T/T_{\text{peak}}$) to calculate the mobility value.[S8–S10] Here, we took the ratio of the peak-to-peak values between ΔT and T instead, which is denoted as $\Delta T_{\text{pp}}/T_{\text{pp}}$. This is because as shown in Figure S3a, the peaks of T and ΔT do not overlap exactly with each other, which means if we took $\Delta T/T$ at the peak of THz pulse, the mobility would be underestimated. Figure S3b shows the mobility values calculated from $\Delta T_{\text{pp}}/T_{\text{pp}}$ and $\Delta T/T_{\text{peak}}$ to demonstrate that by using $\Delta T/T_{\text{peak}}$, and thus not taking into account the shift between the ΔT and T peaks, the mobility is reduced by 25–40%. Compared with the previous mobility measurements taken by Milot *et al.* using $\Delta T/T_{\text{peak}}$ (blue dots),[S10] our mobility values calculated in the same way (green circles) are lower, which is consistent with the fact that our polycrystalline film has a smaller grain size (30–580 nm) than the other sample ($\sim 1\,\mu$m).

2.2 Mobility measured in reflection

In the case of an OPTPS reflection measurement, the $\Delta T/T$ in Equation (S1) is replaced by the reflected THz signal $\Delta R/R$,

$$\frac{\Delta R}{R} \equiv \frac{E_{\text{on}} - E_{\text{off}}}{E_{\text{off}}} = \frac{\Delta E_r}{E_r}. \tag{S16}$$

The solution to Equation (S2) is then modified as[S11]

$$\Delta E_r = -\frac{Z_0 t_1^2 a^2}{2n_1}\Bigg[\int_0^L \exp(2ikz)\Delta\sigma(z)dz + 2r_2\exp(2ikL)\int_0^L \Delta\sigma(z)dz + [r_2\exp(2ikL)]^2\int_0^L \exp(-2ikz)\Delta\sigma(z)dz\Bigg], \tag{S17}$$

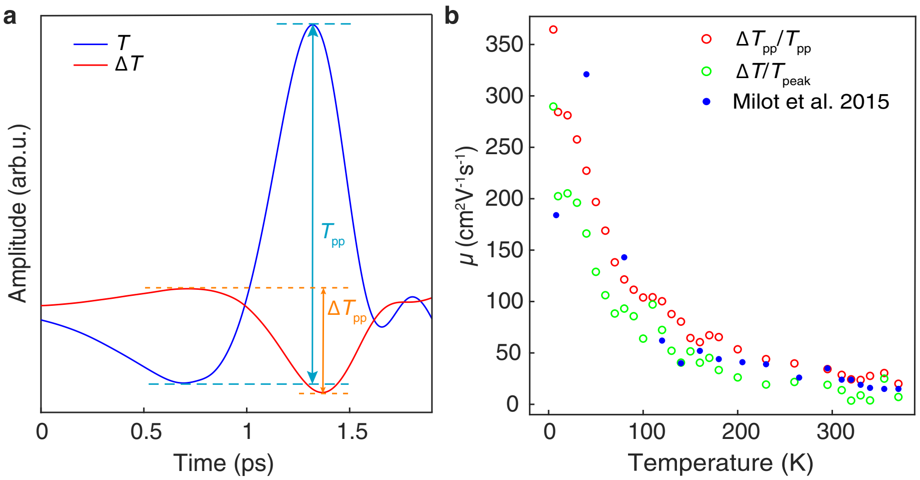

Figure S3: (a) Time-domain traces of T and ΔT of $MAPbI_3$ thin film measured at 5 K, where the blue and red curves represent T and ΔT, respectively. ΔT has been scaled up by a factor of 10 to show the shift between T and ΔT peaks more clearly. (b) Comparison of mobility values calculated using $\Delta T_{\mathrm{pp}}/T_{\mathrm{pp}}$ and $\Delta T/T_{\mathrm{peak}}$, which are represented by the red and green circles, respectively. The blue dots represent the previously reported mobility values of another $MAPbI_3$ thin film sample whose grain size is larger.[S10]

where $t_1 = 2n_1/(n+n_1)$ is the transmission coefficient at the front sample surface. Assuming the incident field E_{inc} is unity, the reflected field E_r is given by

$$E_r = 1 - t_1 = \frac{n_1 - n}{n_1 + n}. \tag{S18}$$

Under the condition of TFA, a simplified expression for $\Delta R/R$ is obtained,[S7]

$$\frac{\Delta R}{R} = -\frac{2a\Delta S}{\varepsilon_0 c(n^2 - n_1^2)}, \tag{S19}$$

where $n_1 = 1$ for the single crystal and $a = 1$ since there is no internal reflection as observed in the thin film sample. Correspondingly, the photoconductivity of the $MAPbI_3$ single crystal is given by

$$\Delta\sigma(\omega) = -\frac{\varepsilon_0 c(n^2 - 1)\alpha}{2}\left(\frac{\Delta R}{R}\right), \tag{S20}$$

where $n = \sqrt{30}$ is the refractive index and $\alpha = 3\times10^5$ cm^{-1} is the absorption coefficient of the $MAPbI_3$ single crystal.[S2,S12] Using Equation (S20), the experimentally measured $-\Delta R/R$ shown in Figure 2b can be converted to photoconductivity $\Delta\sigma$.

According to Equation (S13), the mobility in reflection is expressed as

$$\mu_R = -\frac{\varepsilon_0 c(n^2 - n_1^2)}{2a}\frac{A_{\text{eff}}}{eN}\left(\frac{\Delta R_{\text{pp}}}{R_{\text{pp}}}\right). \tag{S21}$$

This equation also applies to the $MAPbI_3$ single crystal, since its photoexcited layer is still thinner than the THz wavelength required to satisfy the TFA condition. As illustrated in Figure S4b, since we put a sapphire substrate in front of the single crystal to improve the thermal contact, there will be some reflection loss of the pump beam at the air-sapphire and sapphire-crystal interfaces. As a result, the actual intensity of the pump beam at the single crystal surface, I'_{pump}, is less than the initial intensity I_{pump}, which reduces the number of charge carriers given by Equation (S7), such that

$$N = f\varphi\frac{E\lambda}{hc}(1 - R_{\text{pump}})(1 - T_{\text{pump}}), \tag{S22}$$

where $f < 1$ is the power transmission coefficient which can be calculated according to Fresnel equation: in the configuration shown in Figure S4b, $f \approx 0.68$. Finally, the effective mobility measured in reflection is expressed as below,

$$\varphi\mu_R = -\frac{1}{f}\frac{\varepsilon_0 c(n^2 - n_1^2)}{2a}\frac{A_{\text{eff}}hc}{Ee\lambda(1 - R_{\text{pump}})(1 - T_{\text{pump}})}\left(\frac{\Delta R_{\text{pp}}}{R_{\text{pp}}}\right), \tag{S23}$$

where $n_1 = n_{\text{air}}$ and $f = 1$ for the $MAPbI_3$ thin film on quartz (Figure S4a) and $n_1 = n_{\text{sapphire}}$ for a single crystal with sapphire at the front.

Previous discussion has shown that the shift between T and ΔT peaks requires using $\Delta T_{\text{pp}}/T_{\text{pp}}$ instead of $\Delta T/T_{\text{peak}}$ to calculate the mobility. When it comes to calculating the mobility from the reflection measurements, the same principle applies, since both the

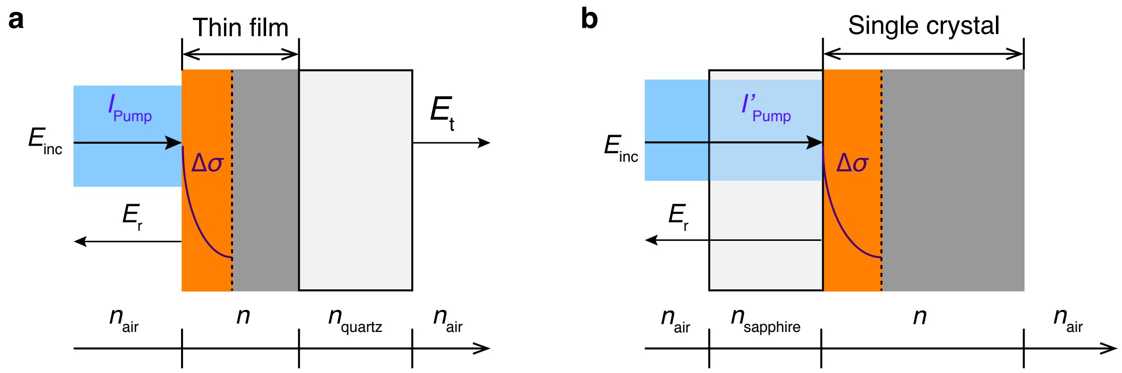

Figure S4: (a) THz and pump pulses are incident normally on the $MAPbI_3$ thin film which is deposited on a quartz substrate. (b) THz and pump pulses are incident normally on the sapphire substrate first which is inserted in front of the $MAPbI_3$ single crystal. The orange area represents the photoexcited layer within the $MAPbI_3$ thin film or single crystal, where the photoconductivity $\Delta\sigma$ follows the Beer-Lambert law. $E_{\rm inc}$, $E_{\rm t}$ and $E_{\rm r}$ represent the incident, transmitted and reflected THz electric field respectively.

$MAPbI_3$ thin film and single crystal exhibit a shift between their R and ΔR peaks as illustrated in Figure S5. While the shift for the thin film is small enough that the mobility calculated using the old method might not deviate so much from the exact value, the much larger shift for the single crystal makes it critical to use $\Delta R_{\rm pp}/R_{\rm pp}$ to extract the mobility value. On top of that, by measuring the traces of the THz pulse and change of THz in the time domain, the noise that comes from the fluctuation of the THz peak is reduced, which gives a better signal-to-noise ratio (SNR).

Figure S6a shows the mobility values of the $MAPbI_3$ thin film measured in reflection using $\Delta R_{\rm pp}/R_{\rm pp}$ and $\Delta R/R_{\rm peak}$, which again shows that our new way of extracting mobility overcomes the issue with the shift between R and ΔR. The less scattered data points of $\Delta R_{\rm pp}/R_{\rm pp}$ (red triangles) also indicate a better SNR. More importantly, in the case of the $MAPbI_3$ single crystal, the mobility measured in reflection using $\Delta R_{\rm pp}/R_{\rm pp}$ is significantly improved, as shown in Figure S6b. Moreover, it is observed that ΔR also shifts with temperature, which makes $\Delta R/R_{\rm peak}$ even less reliable and the temperature-dependence trend represented by the green triangles in Figure S6b less conclusive as well.

Figure S7 shows the mobility of the $MAPbI_3$ thin film measured in transmission and

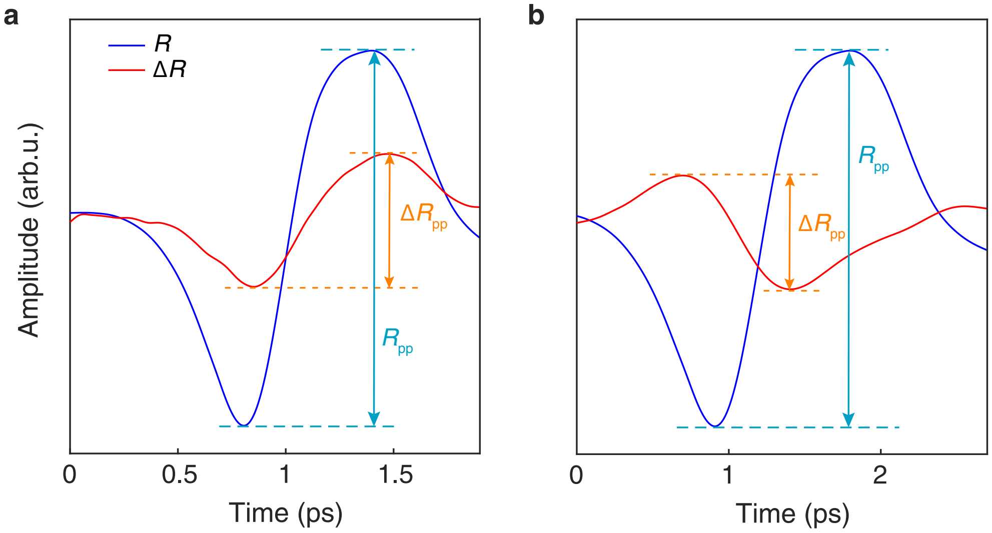

Figure S5: (a) OPTPS measurement of $MAPbI_3$ thin film performed at 5 K. (b) OPTPS measurement of $MAPbI_3$ single crystal performed at 75 K. The blue and red curves represent the reflected THz signal (R) and the change in THz (ΔR) respectively. ΔR has been scaled up by a factor of 40 to show the shift more clearly.

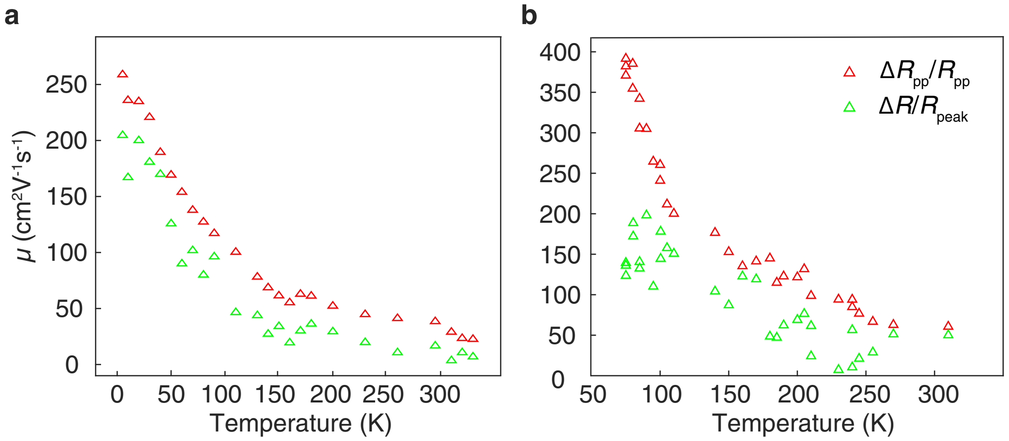

Figure S6: (a) Mobility of $MAPbI_3$ thin film measured in reflection. (b) Mobility of $MAPbI_3$ single crystal measured in reflection. The red and green triangles represent the mobility values calculated using $\Delta R_{\text{pp}}/R_{\text{pp}}$ and $\Delta R/R_{\text{peak}}$ respectively.

reflection, which are calculated by Equations (S15) and (S23), respectively. The consistency between the transmission and reflection measurements verifies the validity of the mobility measurement of the $MAPbI_3$ single crystal, which was done in reflection mode only.

It should be noted that the calculation of mobility using Equations (S15) and (S23) needs

to be done within the low photoexcitation regime where $-\Delta T/T$ (or $-\Delta R/R$) changes linearly with fluence. As illustrated in Figure S8, when the fluence is below $40\,\mu\text{Jcm}^{-2}$, the peak of the transient absorption spectrum, $-\Delta T/T$, increases linearly with fluence and this is the range from which we extract the mobility as reported in the main text. Above $40\,\mu\text{Jcm}^{-2}$, the photoexcitation peak starts to saturate and the mobility value extracted in this range will be an underestimate of the true mobility value.

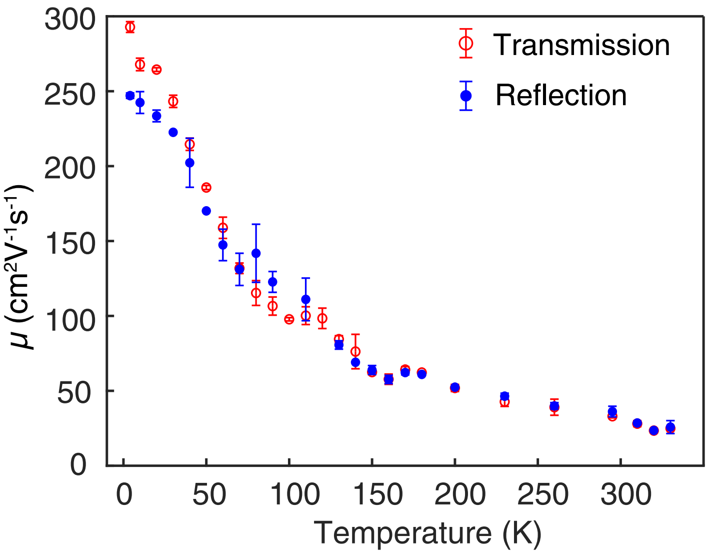

Figure S7: Comparison of the mobility of the $MAPbI_3$ thin film measured in transmission and reflection. The red circles and blue dots represent the transmission and reflection measurements respectively.

2.3 Literature values of electrical mobility in $MAPbI_3$

As mentioned in the main text, a wide range of electrical mobilities has previously been reported for $MAPbI_3$ single crystals and thin films using different experimental methods, which is summarised in Table S1. Depending on which experimental method is used, the measured mobility can refer to the mobility of a single carrier species (i.e. either electron mobility μ_e or hole mobility μ_h), or the sum of both μ_e and μ_h.

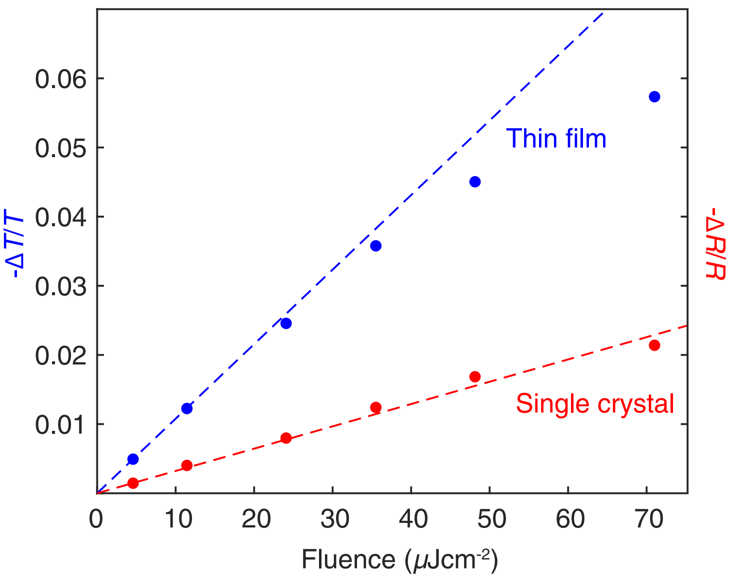

Figure S8: Peak of transient absorption spectrum measured at different fluences. The blue and red dots represent the experimental data from the $MAPbI_3$ thin film and single crystal respectively. The blue and red dashed lines represent the corresponding linear fits in the low-fluence regime (below $40\,\mu Jcm^{-2}$), where the mobility value is extracted.

Table S1: Comparison of electrical mobilities of $MAPbI_3$ single crystals and polycrystalline thin films measured using different experimental methods. $\mu = \mu_e + \mu_h$ denotes the total charge-carrier mobility, where μ_e represents the electron mobility and μ_h represents the hole mobility.

Method	Room-temperature mobility ($cm^2V^{-1}s^{-1}$)	Ref.
Hall effect	$\mu_e = 66$ (single crystal)	S13
	$\mu_e = 25$, $\mu_h = 105$ (single crystal)	S14
Space-charge limited current	$\mu_h = 164$ (single crystal)	S14
(SCLC)	$\mu_h = 2.5$ (single crystal)	S15
	$\mu_h = 67$ (single crystal)	S16
Time-of-flight	$\mu_e = 24$ (single crystal)	S14
(TOF)	$\mu_e = 135$, $\mu_h = 90$ (single crystal)	S17
Field-effect transistor characterization	$\mu_e = 0.5$ (thin film)	S18
	$\mu_e = 0.7$, $\mu_h = 1.5$ (single crystal)	S19
Microwave conductivity	$\mu = 115$ (single crystal)	S20
	$\mu = 30$ (thin film)	S21
	$\mu = 29$ (thin film)	S22
THz conductivity	$\mu = 600$ (single crystal)	S23
	$\mu = 8$ (thin film)	S9
	$\mu = 27$ (thin film)	S24
	$\mu = 35$ (thin film)	S10

3 Effect of grain-boundary scattering on electrical mobility

3.1 Theoretical modeling

Since one crucial difference between the $MAPbI_3$ polycrystalline thin film and the single crystal is the existence of grain boundaries, it is important to take into account their effect on the charge-carrier mobility as a function of temperature. This was done through the *ab initio* transport calculation of carrier mean free path $\lambda_{\text{transport}}$ in Equation (3) of the main text. However, it is also interesting to look at the mean free path calculated at the most representative energy, which we determined in an earlier work[S25] to be $(3/2)k_{\mathrm{B}}T$ away from the band edge. This is the energy at which the carrier contributes most to the transport properties. The carrier mean free path thus becomes

$$\lambda_{(3/2)k_{\mathrm{B}}T} = \frac{1}{3N}\sum_{\alpha,n\boldsymbol{k}} |\mathbf{v}^{\alpha}_{n\boldsymbol{k}}|\tau_{n\boldsymbol{k}} \quad \text{for} \quad n\boldsymbol{k} \in \frac{3}{2}k_{\mathrm{B}}T - \Delta \leq \varepsilon_{n\boldsymbol{k}} \leq \frac{3}{2}k_{\mathrm{B}}T + \Delta, \tag{S24}$$

where $\mathbf{v}_{n\boldsymbol{k}}$ is the carrier velocity and $\tau_{n\boldsymbol{k}}$ is the electron-phonon lifetime for a state of band n and momentum $\boldsymbol{k}$. The mean free path average for electrons and holes is performed for states $n\boldsymbol{k}$ such that their energy is within $\Delta = 1.4\,\text{meV}$ of the dominant energy $(3/2)k_{\mathrm{B}}T$ from the band edge. The comparison between the transport mean free path and the mean free path computed at the most representative energy is shown in Figure S9 with solid and dashed lines, respectively. The most representative mean free path closely follows the transport one but slightly underestimates it, leading to a predicted grain size of around 60 nm. If one uses the correct transport mean free path, the predicted grain size is instead around 100 nm as can be seen in Figure S10 where various other grain sizes are also shown for comparison. We highlight that the grain size g is the only fitting parameter since the probability of reflection is fixed to $R = 0.5$ and can be used for other perovskite materials.[S26,S27] However, for

completeness, we show in Figure S11 the change of room-temperature electrical mobility as a function of grain size g and reflection probability R. It is noted that the mobility depends moderately on those two parameters. Specifically, the mobility changes from $10\,\mathrm{cm^2V^{-1}s^{-1}}$ with $g = 25\,\mathrm{nm}$ and $R = 0.75$, to $70\,\mathrm{cm^2V^{-1}s^{-1}}$ with $g = 200\,\mathrm{nm}$ and $R = 0.25$.

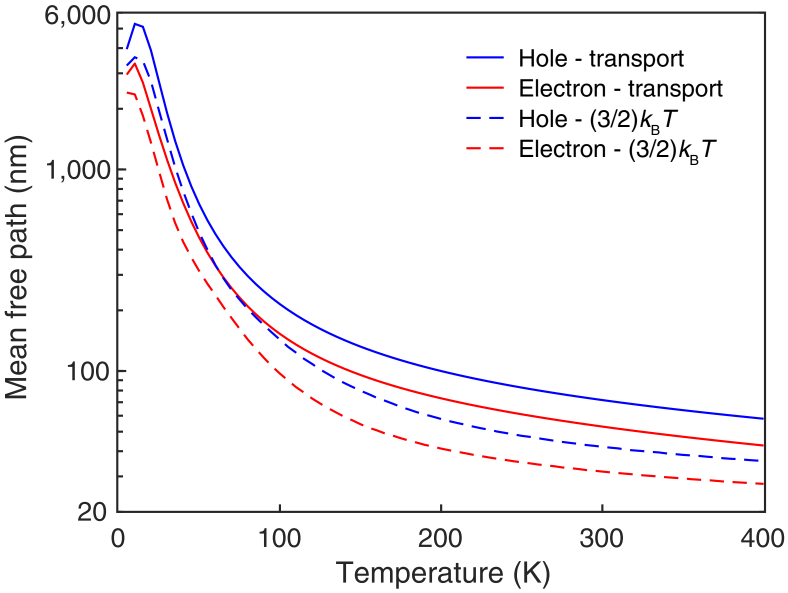

Figure S9: Comparison between the hole and electron mean free paths. The solid blue and red lines represent the ab initio transport mean free path for the hole and electron respectively. The dashed blue and red lines represent the mean free path calculated for the most relevant energy $(3/2)k_\mathrm{B}T$.

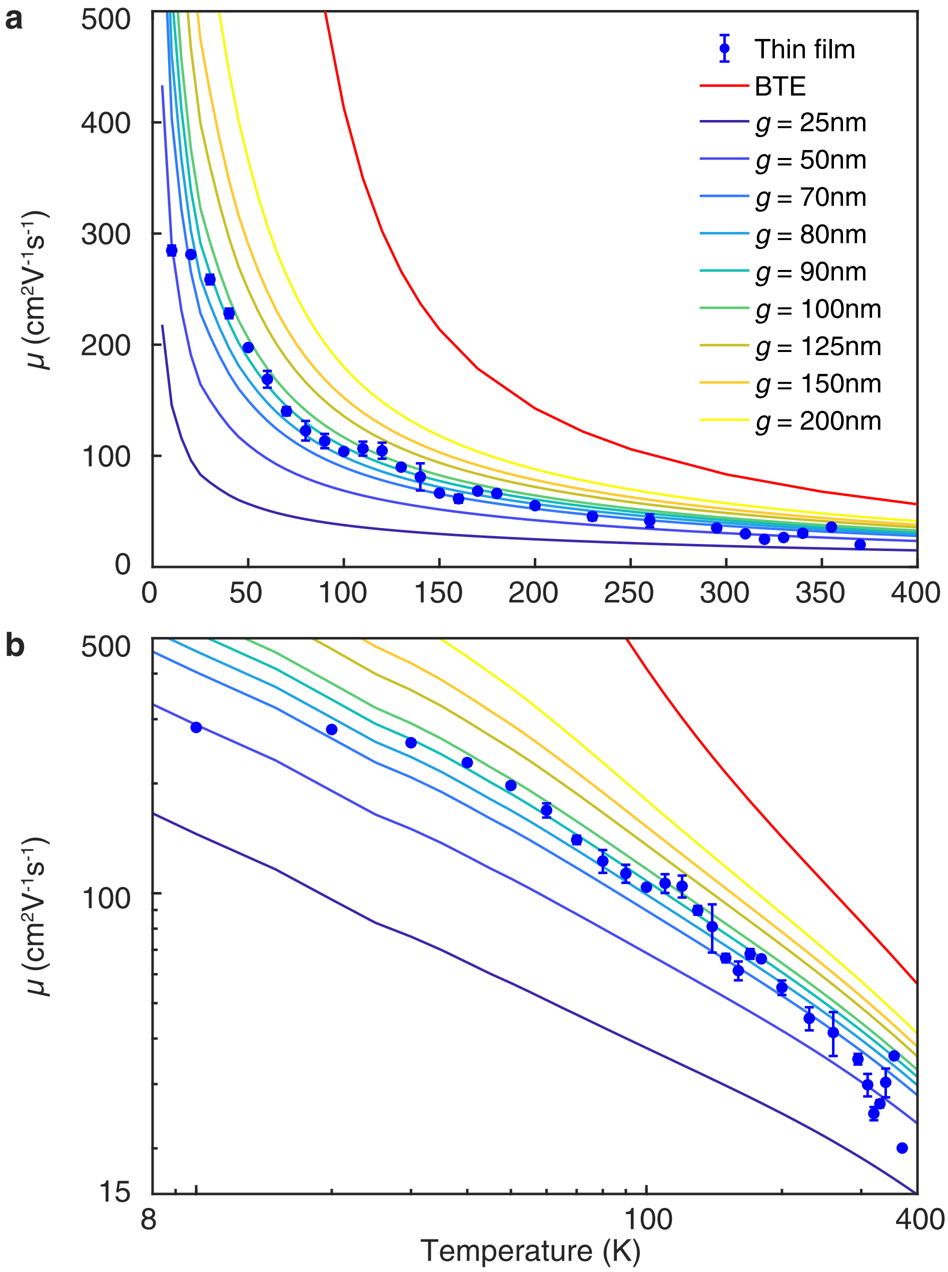

Figure S10: (a) Intrinsic carrier mobility of electrons and holes computed with the Boltzmann transport equation (red) compared with the mobility resulting from the addition of grain-boundary scattering effects using the ab initio transport mean free path. Experimental thin film results represented by the blue dots are reproduced by a grain size of 100 nm. (b) Same mobility plot on a log-log scale to indicate the effect of grain size on the temperature-dependent exponent.

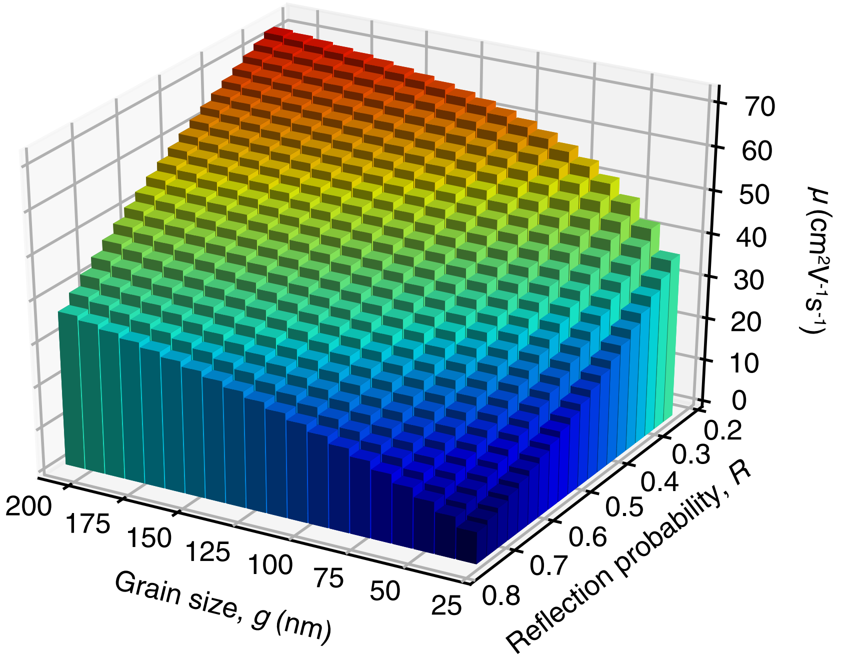

Figure S11: Room-temperature mobility of $MAPbI_3$ as a function of grain size g and reflection probability R.

3.2 Grain-size measurement of polycrystalline $MAPbI_3$ thin film

In order to measure the grain size in the $MAPbI_3$ thin film, a top-down scanning electron microscope (SEM) image was taken over an area of $9\,\mu\text{m}\times 8\,\mu\text{m}$ as shown in Figure S12a. An imaging processing program (ImageJ[S28]) was used to analyze the lateral size of each grain within this area, and a histogram showing the distribution of grain sizes is shown in Figure S12b, from which the average grain size is found to be (580 ± 212) nm.

Since the SEM only gives the upper limit of the grain size and some sub-grain features might be hidden,[S29] we performed an X-ray diffraction (XRD) measurement on the thin film to obtain a better estimate of the grain size. Figure S12c shows the Gaussian-fit to the XRD spectrum given in Figure 1f in the main text, from which the width of each diffraction peak is extracted and used for calculating the grain size g according to Scherrer equation,[S30]

$$g = \frac{K\lambda_{\text{source}}}{\beta \cos\theta}, \tag{S25}$$

where $K = 0.93$ is the shape constant, $\lambda_{\text{source}} = 0.154\,\text{nm}$ is the wavelength of the X-ray source, β is the FWHM of each diffraction peak and θ is the Bragg angle. The resultant grain size averaged over three diffraction peaks, (110), (220) and (330), is $(31 \pm 10)\,\text{nm}$.

As mentioned in the main text, the XRD peaks might have been broadened further due to strain effects, which places a lower bound on the grain size. Therefore, based on the SEM and XRD measurements, we estimate the grain size of the $MAPbI_3$ thin film to be between 30–580 nm.

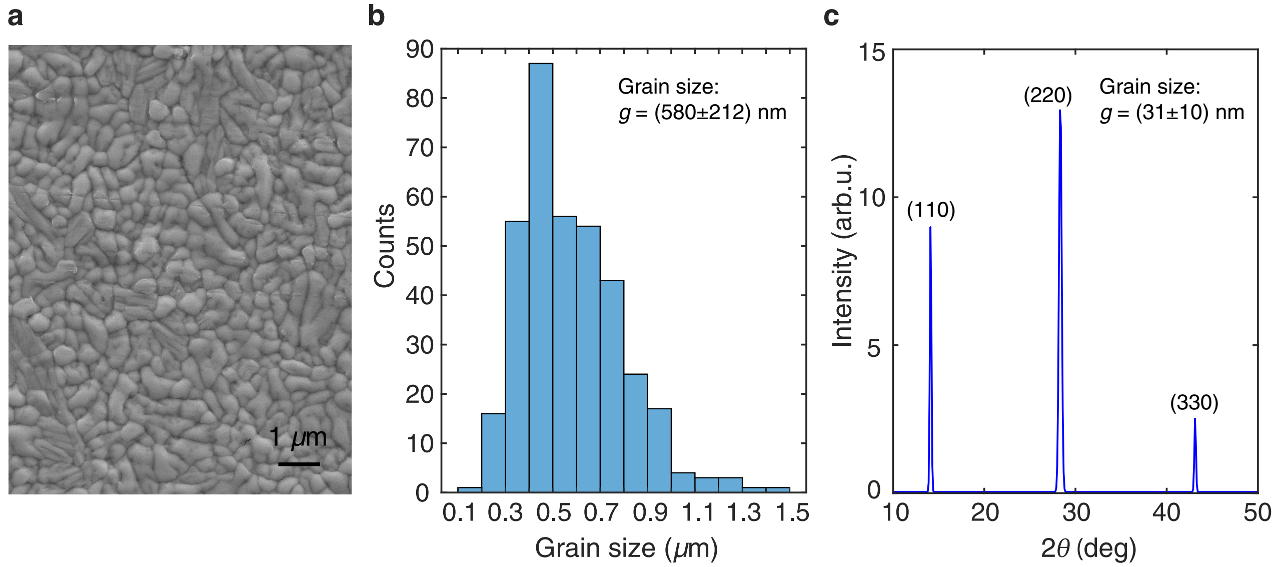

Figure S12: (a) SEM image of the $MAPbI_3$ thin film in an area of $9\,\mu\text{m} \times 8\,\mu\text{m}$. (b) Histogram of the distribution of grain sizes at the surface of the thin film. (c) XRD spectrum of the thin film where each diffraction peak is fitted with a Gaussian distribution function to extract the FWHM.

4 Significance of photon-reabsorption effects

4.1 Charge-carrier dynamics analysis

As described in the main text, we incorporate the effect of photon reabsorption and carrier diffusion into the charge-carrier dynamics of $MAPbI_3$ to extract the intrinsic recombination rates according to Equation (1),

$$\frac{\partial n}{\partial t} = D\frac{\partial^2 n}{\partial z^2} + G - k_1 n - k_2 n^2 - k_3 n^3,$$

with the diffusion coefficient D determined by

$$D = \frac{\mu k_\mathrm{B} T}{e}, \tag{S26}$$

where μ is the electrical mobility, k_B is the Boltzmann constant and T is the sample temperature. The monomolecular recombination rate k_1 was obtained from a time-resolved photoluminescence (TRPL) measurement which is shown in Figure S13. According to the stretched exponential fit to the transient measured at the lowest photoexcitation fluence, the single crystal shows a longer average charge-carrier lifetime (54 ns) than the thin film (12 ns). The Auger recombination rate k_3 was neglected (i.e. $k_3 = 0$) in the low-fluence regime.

In order to solve Equation (1), a self-absorption (photon-reabsorption) model[S31] was used, in which the sample was divided into multiple thin slices (typically 2-nm thick) with the application of a finite-difference-time-domain method. While the total thickness of the thin-film $MAPbI_3$ is around 300–600 nm, the thickness of the photoexcited layer in the thick single crystal is approximated as a few microns. The charge-carrier density $n(t, z)$ at each point in the sample was updated every 1 ps under diffusion, from which the number of photons generated via bimolecular recombination was determined. Those photons can then be reabsorbed by the sample, producing new electron-hole pairs and delaying the charge-

carrier recombination process. The update of $n(t, z)$ was repeated for a total time period of 1600 ps, covering the entire time window shown in Figure 2. The photoconductivity was then determined by $\Delta\sigma(t) = n(t)e\mu$ where $n(t)$ is the sum of charge-carrier densities across all of the slices, which was fitted globally to the experimentally measured photoconductivity decay dynamics shown in Figure 2. As a result, the bimolecular recombination rate k_2 was extracted as 2.6×10^{-10} cm^{3}s^{-1} for the thin film and 8.7×10^{-10} cm^{3}s^{-1} for the single crystal.

To demonstrate that our photon-reabsorption model describes the charge-carrier dynamics in $MAPbI_3$ more accurately and is able to extract the intrinsic bimolecular recombination rate, we compare its result with that obtained from a simple model which neglects the effect of photon reabsorption and is described by the following rate equation,

$$\frac{\partial n}{\partial t} = G' - k_1 n - k_2 n^2 - k_3 n^3, \tag{S27}$$

where G' refers to the charge-carrier generation rate which arises only from the initial photoexcitation. Since the effect of photon reabsorption is neglected in this simple model, the diffusion term given in Equation (1) in the main text is omitted. Figure S14 shows the theoretical fit to the photoconductivity decay dynamics using this simple model. While the fit to the $MAPbI_3$ thin film data looks reasonably good, the fit to the $MAPbI_3$ single crystal data is much worse than that obtained from the photon-reabsorption model shown in Figure 2. This is because the single crystal is much thicker than the thin film, which makes the effect of photon reabsorption much more prominent and the neglect of photon reabsorption can lead to inaccurate analysis of the charge-carrier dynamics. In particular, as pointed out by Crothers et al.,[S31] photon reabsorption can mask the intrinsic charge-carrier recombination rate significantly, which makes the simple model governed by Equation (S27) invalid.

Table S2 lists the charge-carrier recombination rates obtained from the photon-reabsorption model and the simple model, governed by Equations (1) and (S27) respectively. It is clear that while the photon-reabsorption model is able to obtain similar bimolecular recombina-

tion rates between the $MAPbI_3$ thin film and single crystal as expected, the simple model results in very different values of k_2 by two orders of magnitude. In the case of the $MAPbI_3$ single crystal, due to the significant effect of photon absorption, the charge-carrier decay dynamics (Figure S14b) appears to decay slower than that of the thin film (Figure S14a), which results in a smaller bimolecular recombination rate if using the simple model. However, the recombination rate as an intrinsic property of the material should be independent of the sample thickness, which means the value of k_2 extracted from the simple model has been masked by the effect of photon reabsorption. Therefore, it is crucial to take into account the effect of photon reabsorption when analysing the charge-carrier dynamics, especially for thick samples such as a single crystal.

Table S2: Comparison of bimolecular recombination rates, k_2, extracted from the photon-reabsorption model and the simple model (no photon absorption). The monomolecular recombination rates, k_1, were obtained from the TRPL measurements.

	Photon reabsorption	No photon reabsorption
k_1 [s^{-1}] (extracted from TRPL)	$k_{1,\text{film}} = 8.3 \times 10^7$<br>$k_{1,\text{crystal}} = 1.85 \times 10^7$	
$k_{2,\text{film}}$ [$cm^3 s^{-1}$]	2.6×10^{-10}	1.17×10^{-9}
$k_{2,\text{crystal}}$ [$cm^3 s^{-1}$]	8.7×10^{-10}	3.65×10^{-11}

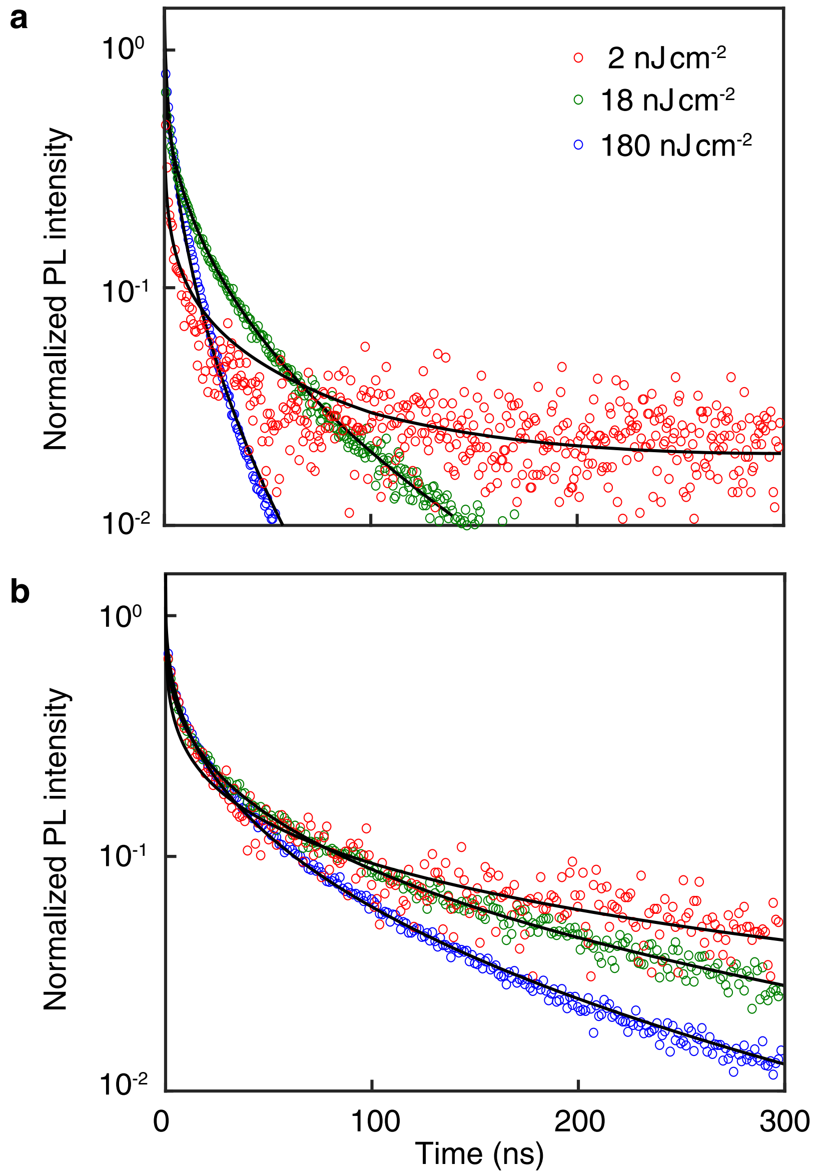

Figure S13: Time-resolved photoluminescence (TRPL) transients of $MAPbI_3$ (a) thin film and (b) single crystal. The solid curves represent the stretched exponential fits to the experimental data (open circles), from which k_1 is extracted.

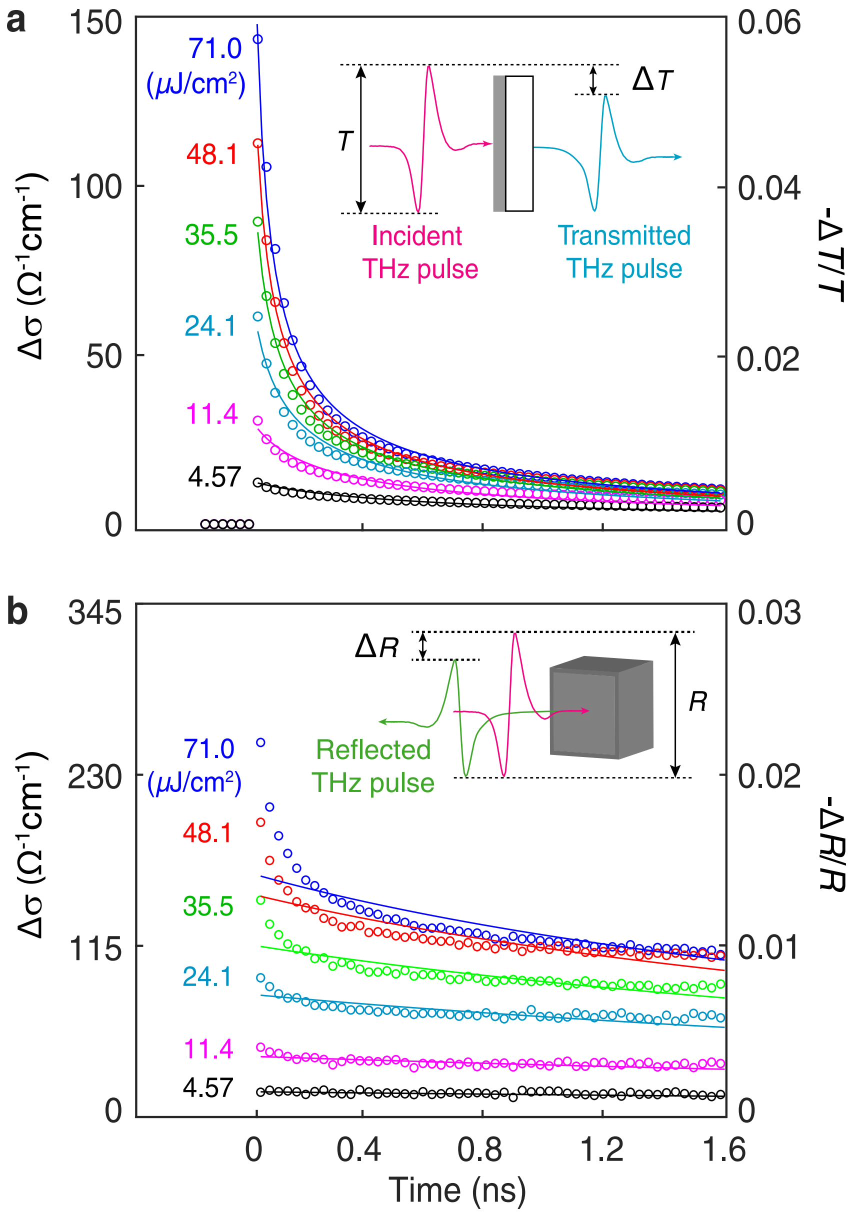

Figure S14: Photoconductivity decay dynamics of (a) $MAPbI_3$ thin film measured in transmission and (b) $MAPbI_3$ single crystal measured in reflection. The circles represent the experimental data identical to what is shown in Figure 2 in the main text. The curves represent the theoretical fits obtained from the simple model governed by Equation (S27).

4.2 Charge-carrier diffusion length calculation

Having obtained the charge-carrier mobility and recombination rates, we can calculate the carrier diffusion length according to Equation(2),

$$L_{\mathrm{D}}(n) = \sqrt{\frac{D}{R(n)}},$$

where D is the diffusion constant given by Equation (S26) and R represents the total recombination rate of k_1, k_2 and k_3, which is expressed as

$$R = n^2 k_3 + n k_2 + k_1, \tag{S28}$$

where n is the charge-carrier density, which depends on the photoexcitation intensity and has a significant effect on carrier diffusion length. Figure S15 shows the diffusion lengths as a function of carrier density, which were calculated at different values of k_1, with the bimolecular recombination rates, $k_{2,\mathrm{film}} = 2.6 \times 10^{-10}\,\mathrm{cm^3 s^{-1}}$ and $k_{2,\mathrm{crystal}} = 8.7 \times 10^{-10}\,\mathrm{cm^3 s^{-1}}$, extracted from the photon-reabsorption model. The electrical mobilities used for thin film and single crystal are 33 and $59\,\mathrm{cm^2 V^{-1} s^{-1}}$ respectively, which were obtained from room-temperature photoconductivity measurement (Figure 2 in the main text). The arrows indicate the diffusion lengths obtained at certain carrier densities, $n_{\mathrm{AM1.5}}$, corresponding to 1 sun illumination ($1\,\mathrm{kWm^{-2}}$ AM1.5-filtered), which is the usual operating condition for perovskite solar cells. According to Ref. S32, under 1 sun illumination with the assumption of no charge extraction, the carrier density is estimated by setting $\partial n/\partial t = 0$ in Equation (1) given in the main text. The Auger recombination k_3 and the diffusion term $D(\partial^2 n/\partial z^2)$ are neglected for a simplistic approximation. The resultant quadratic equation can be solved with known charge-carrier generation rate G, which is determined from the following equation,

$$G = \frac{1}{hc} \int f_{\mathrm{solar}}(\lambda)\alpha(\lambda)e^{-\alpha(\lambda)z}\lambda d\lambda, \tag{S29}$$

where h is the Planck constant, c is the speed of light in vacuum, z is the film depth and $\alpha(\lambda)$ is the absorption coefficient of $MAPbI_3$ at wavelength λ. f_{solar} is the ASTM G173-03 Global Tilt reference spectrum for the solar spectral irradiance distribution, which satisfies $\int f_{\mathrm{solar}}(\lambda)d\lambda = 1\,\mathrm{kWm^{-2}}$. An average value $\langle G \rangle = 4 \times 10^{21}\,\mathrm{cm^{-3}s^{-1}}$ is extracted for a typical $MAPbI_3$ thin film,[S32] from which the carrier density $n_{\mathrm{AM1.5}}$ and diffusion length can be determined.

As shown in Table S3, the diffusion lengths of the $MAPbI_3$ single crystal extracted from the simple model is significantly longer than that of the thin film, whereas the difference in diffusion lengths obtained from the photon-reabsorption model is much smaller. In particular, when k_1 is less than $10^7\,\mathrm{s^{-1}}$, such difference is more prominent, since the difference in $n_{\mathrm{AM1.5}}$ between the photon-reabsorption model and the simple model is larger as shown in Figure S16.

Table S3: Carrier diffusion length calculated from the photon-reabsorption model and the simple model under 1 sun illumination for $MAPbI_3$ thin film ($L_{\mathrm{D,film}}$) and single crystal ($L_{\mathrm{D,crystal}}$). Different k_1 values were used for demonstrating the effect of charge-carrier density on diffusion length. The bottom cell shows the diffusion lengths calculated using k_1 extracted from the TRPL measurements.

	Photon reabsorption	No photon reabsorption
	$k_1 = 0$	
$L_{\mathrm{D,film}}$ [μm]	9.1	6.2
$L_{\mathrm{D,crystal}}$ [μm]	9.1	20
	$k_1 = 10^5\,\mathrm{s^{-1}}$	
$L_{\mathrm{D,film}}$ [μm]	8.85	6.16
$L_{\mathrm{D,crystal}}$ [μm]	8.85	18.6
(TRPL)	$k_{1,\mathrm{film}} = 8.3 \times 10^7\,\mathrm{s^{-1}}$<br>$k_{1,\mathrm{crystal}} = 1.85 \times 10^7\,\mathrm{s^{-1}}$	
$L_{\mathrm{D,film}}$ [μm]	1.0	1.0
$L_{\mathrm{D,crystal}}$ [μm]	2.83	2.85

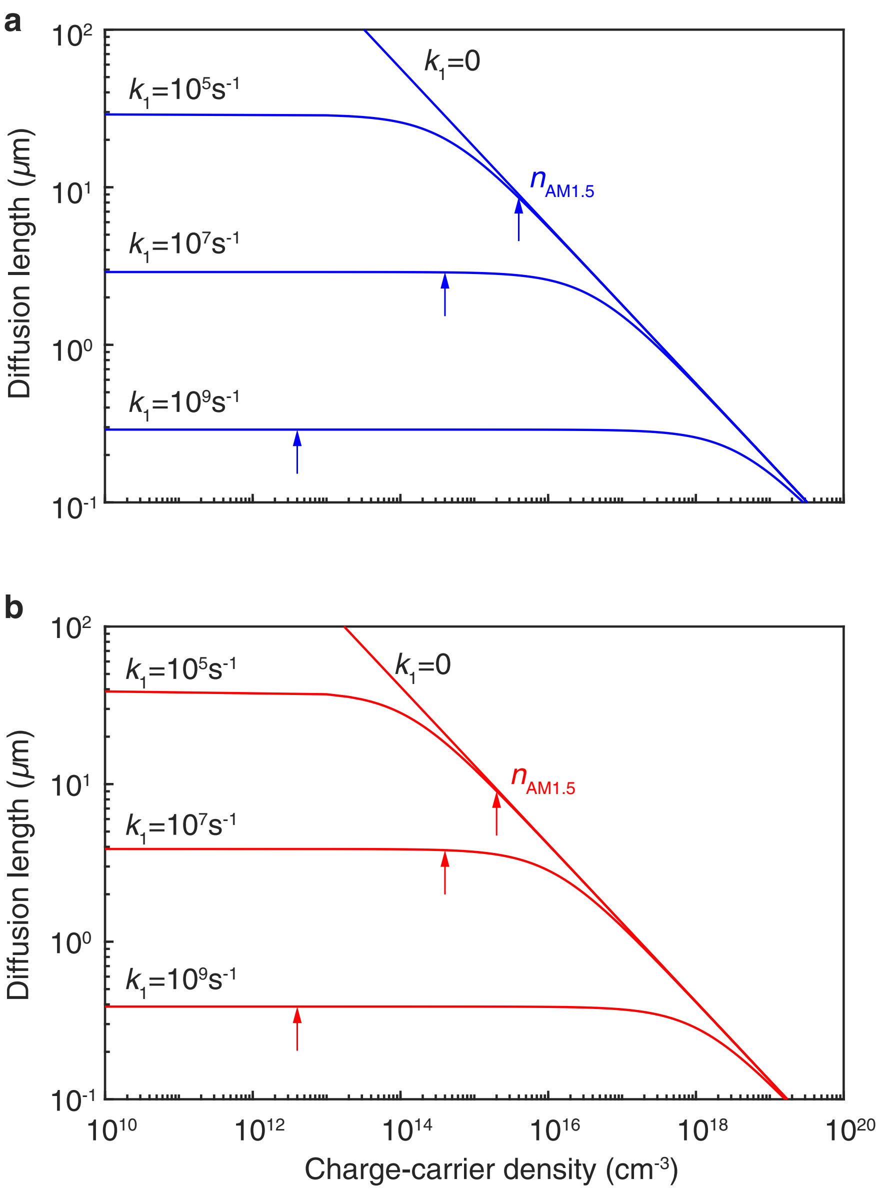

Figure S15: Diffusion length of (a) $MAPbI_3$ thin film and (b) single crystal as a function of carrier density with different values of k_1. The photon-reabsorption model was used for extracting the values of k_2. The arrows indicate the carrier densities corresponding to 1 sun illumination, $n_{AM1.5}$.

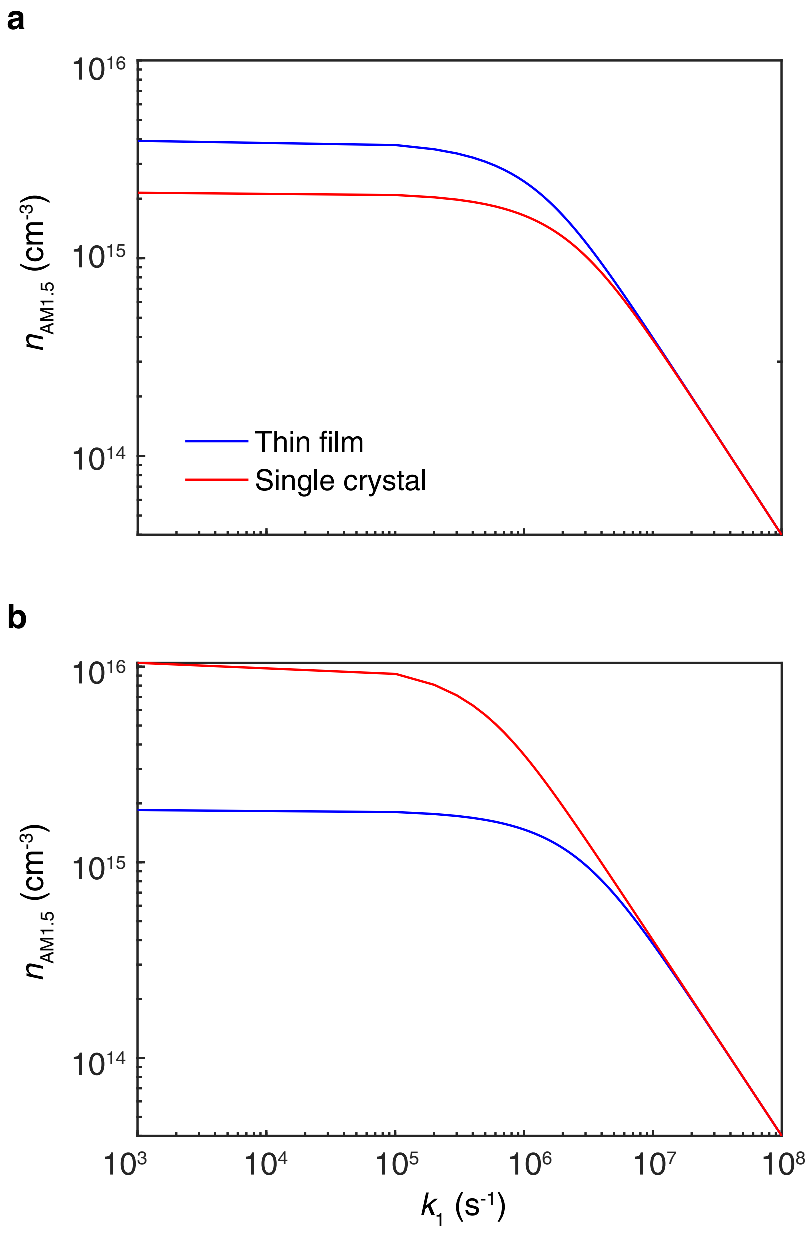

Figure S16: Charge-carrier density $n_{\mathrm{AM1.5}}$ plotted as a function of k_1, with k_2 values extracted from (a) the photon-reabsorption model and (b) the simple model. The blue and red curves represent the results for the $\mathrm{MAPbI_3}$ thin film and single crystal respectively.

4.3 Photoconductivity decay dynamics of thin film measured in reflection

We also measured the photoconductivity decay dynamics of $MAPbI_3$ thin film at room temperature in reflection (Figure S17) to show its consistency with the transmission measurement described in the main text (Figure 2a). According to Equation (S23), we extracted the electrical mobility of thin film in reflection as $(29 \pm 3)\,\mathrm{cm^2V^{-1}s^{-1}}$, which agrees with the value measured in transmission, i.e. $(33 \pm 2)\,\mathrm{cm^2V^{-1}s^{-1}}$. The bimolecular recombination rate of thin film measured in reflection is $2.9 \times 10^{-10}\,\mathrm{cm^3s^{-1}}$, which is also consistent with the transmission measurement.

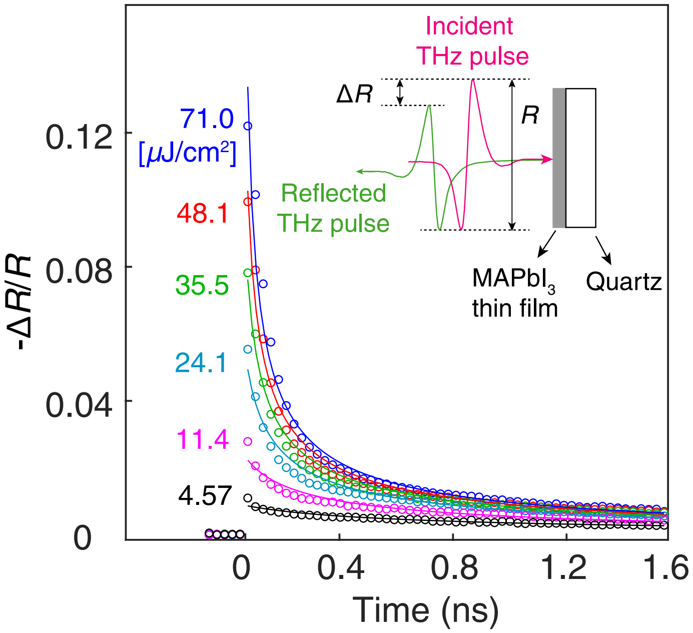

Figure S17: Photoconductivity decay dynamics of $MAPbI_3$ thin film measured in reflection at room temperature. The photoexcitation fluence ranges from 4.57 to 71.0 $\mu\mathrm{Jcm^{-2}}$.

5 PL-facilitated temperature correction of $MAPbI_3$ single crystal

To measure the mobility of the single crystal as a function of temperature, we mounted the crystal on a cold-finger cryostat (Oxford Instruments, MicrostatHe) to get it down to low temperature. Unlike the thin film, it was much more challenging to cool down the single crystal to low temperature due to its poor thermal conductivity. Therefore, we inserted a sapphire disc (2 mm in thickness and 13 mm in diameter) between the crystal and the sample holder to improve the thermal contact and dissipate the heat generated by the pump beam more efficiently. As a result, we were able to cool down the crystal to a much lower temperature. However, because the temperatures between the cold-finger cryostat and the crystal can be slightly different, the true temperature of the crystal could not be determined accurately. Thankfully, with the help of a gas-exchange cryostat (Oxford Instruments, OptistatCF2), the temperature of the single crystal could be measured more accurately and reach the minimum of 5 K. This is because as the single crystal was surrounded by helium gas, it did not have the problem with thermal contact. Therefore, we measured the PL spectra of the single crystal in the gas-exchange cryostat at various temperatures and used this set of PL spectra as a reference to correct the crystal temperature measured using the cold-finger cryostat.

Figures S18 and S19 show the PL spectra of the single crystal measured in the gas-exchange cryostat and cold-finger cryostat, respectively. Figure S20 shows the PL of the thin film measured in the cold-finger cryostat. By fitting a two-Gaussian function, the PL spectra were resolved into two peaks, P1 and P2, which eventually converge to the same wavelength at high temperature.

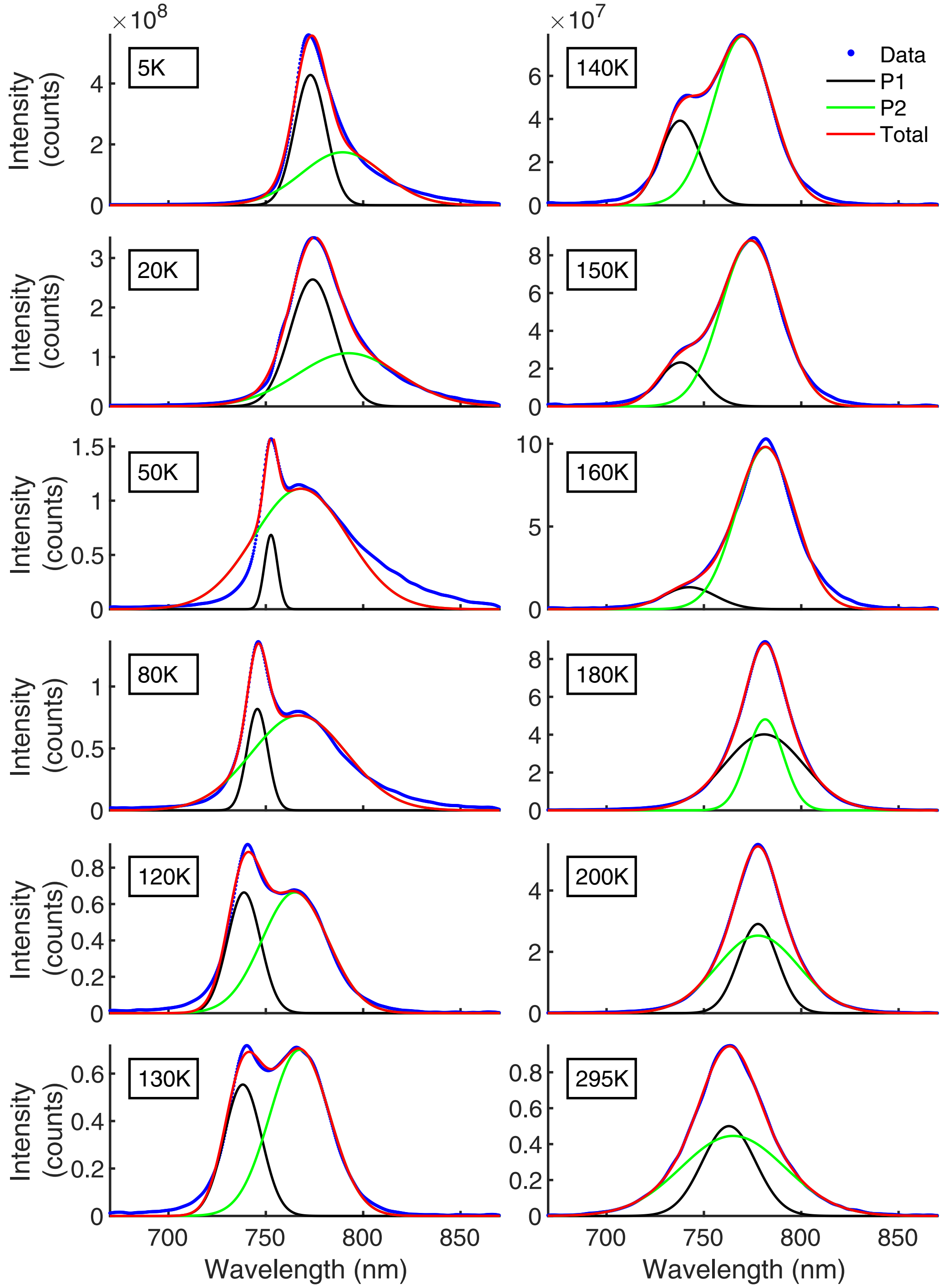

Figure S18: PL spectra of the single crystal measured by gas-exchange cryostat at different temperatures. The crystal was photoexcited by a pulsed laser at 398 nm with a 10 kHz repetition rate and a fluence of $0.2\,\mu Jcm^{-2}$. The blue curves represent the PL data, whereas the red curve represents the Gaussian fit which consists of two peaks represented by the black curve and the green curve, respectively.

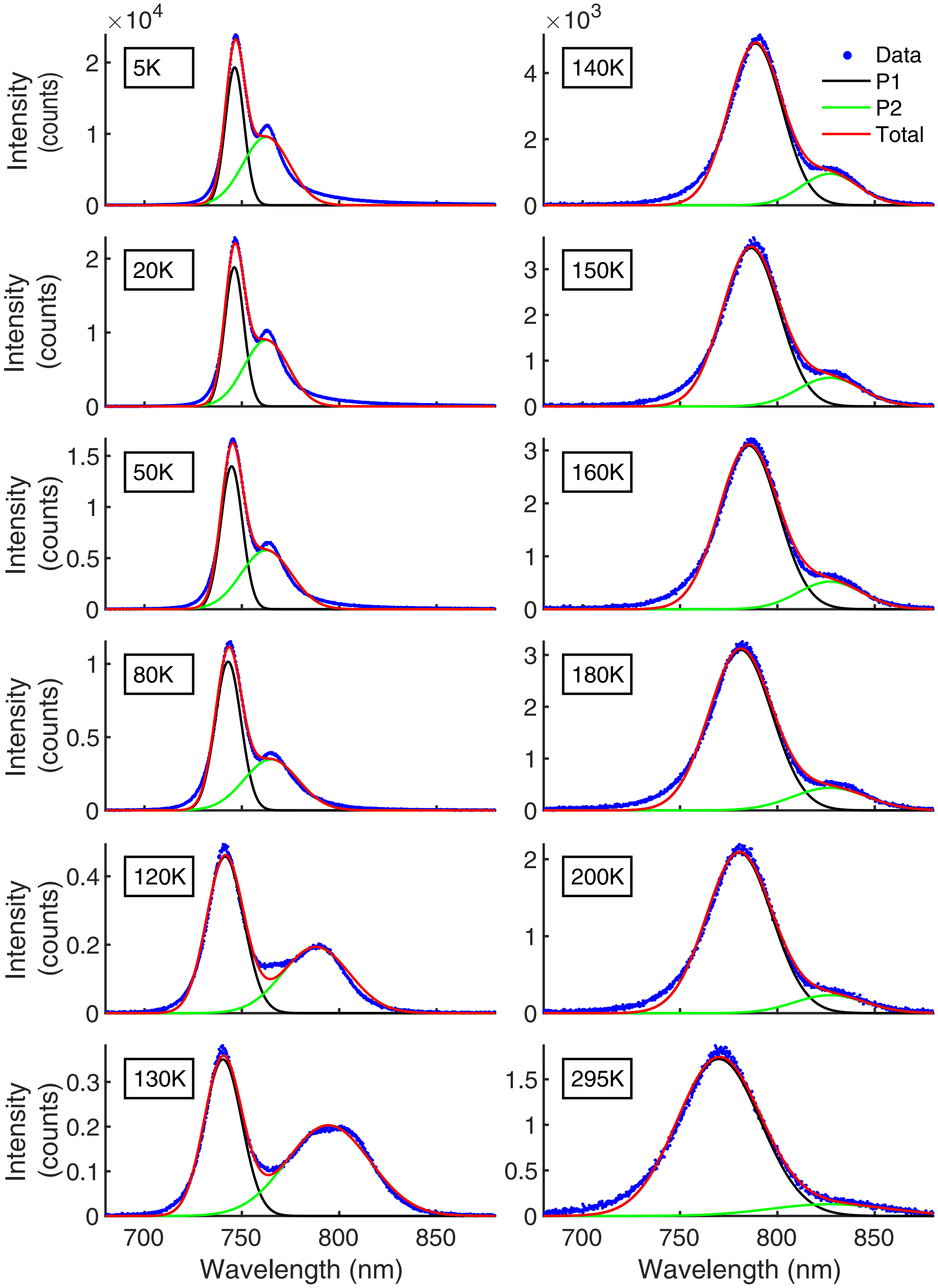

Figure S19: PL spectra of the single crystal measured by cold-finger cryostat at different temperatures. The crystal was photoexcited by a pulsed laser at 400 nm with a 5 kHz repetition rate and a fluence of 30.8 μJcm^{-2}. The blue dots represent the PL data, whereas the red curve represents the Gaussian fit which consists of two peaks represented by the black curve and the green curve, respectively.

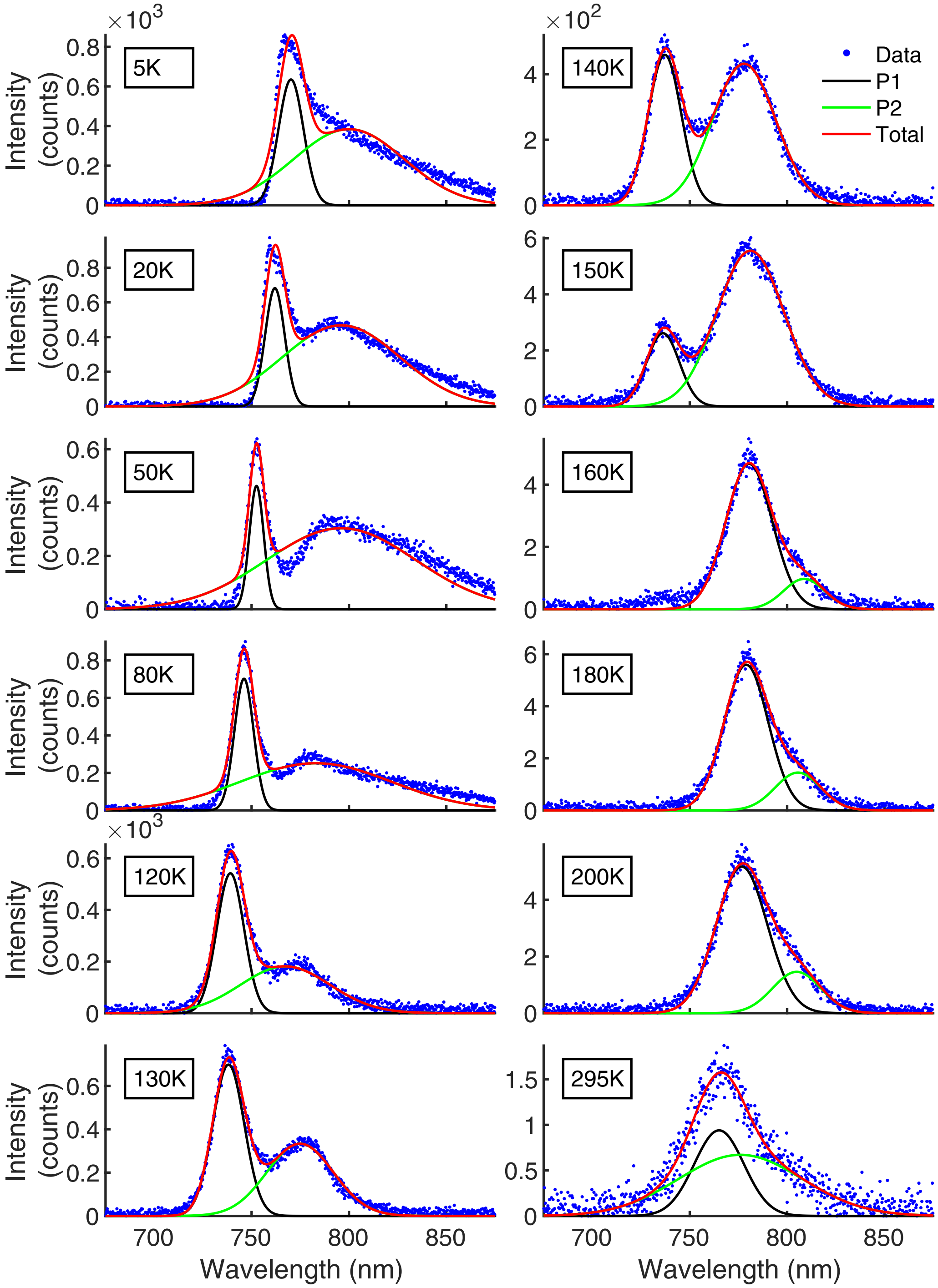

Figure S20: PL spectra of the thin film measured by cold-finger cryostat at different temperatures. The thin film was photoexcited by a pulsed laser at 400 nm with a 5 kHz repetition rate and a fluence of 30.8 μJcm^{-2}. The blue dots represent the PL data, whereas the red curve represents the Gaussian fit which consists of two peaks represented by the black curve and the green curve, respectively.

Figure S21 shows the central wavelengths of P1 and P2 for the single crystal as a function of temperature, which was measured by the gas-exchange cryostat. There is an abrupt redshift of the peak wavelengths at the orthorhombic-tetragonal phase transition at 160 K, which is consistent with what has been reported previously on $MAPbI_3$ thin films.[S10] Moreover, both P1 and P2 show similar temperature dependence throughout the entire temperature range, although the phase shift of P1 at 160 K is more significant compared to the gradual redshift of P2 at temperatures 115–165 K.

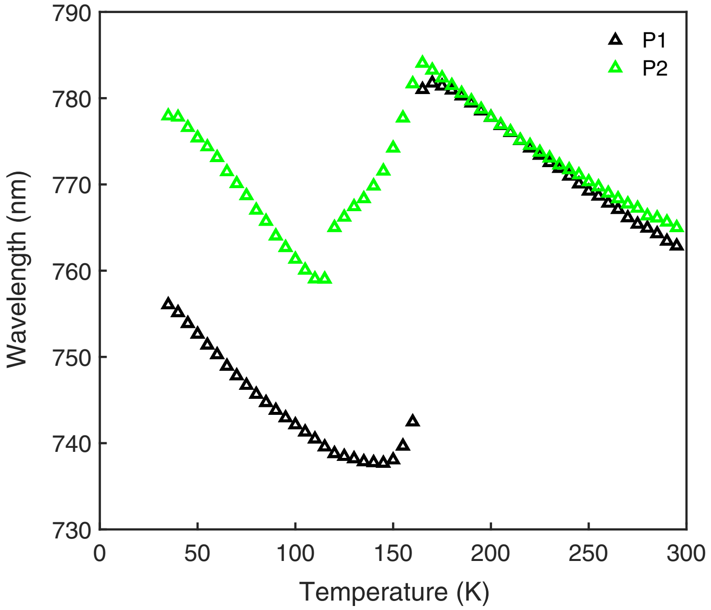

Figure S21: Central wavelength of single crystal's PL spectrum measured by the gas-exchange cryostat as a function of temperature. The black and green triangles represent the central wavelengths of P1 and P2 respectively.

Since P1 and P2 converge to the same wavelength above 160 K and P1 is more prominent in the cold-finger cryostat measurement of the single crystal (see Figure S19), we decided to take P1 as the reference PL peak for correcting the single crystal's temperature. As shown in Figure S22a, before temperature correction, P1 of the single crystal measured by the cold-finger cryostat (pink triangle) deviates from the gas-exchange measurement (blue triangles), whereas the thin film measurement (black circles) shows a good agreement due to its good thermal contact with the cold-finger cryostat. In Figure S22b, the cold-finger

cryostat temperature is matched with the gas-exchange measurement, from which we can determine the single crystal's real temperature accurately. One might have noticed that the relative intensity of P1 and P2 is slightly different between the gas-exchange measurement (Figure S18) and the cold-finger measurement (Figure S19) of the single crystal at 140 K and 150 K. This is attributed to the different excitation fluence between these two sets of measurements, which differs by two orders of magnitude. In addition, the extra sapphire substrate in the cold-finger cryostat configuration may also affect the photon reabsorption and emission in the $MAPbI_3$ single crystal, which results in different intensity ratios between P1 and P2. Nevertheless, the more crucial feature in the PL spectra measurement is the peak positions of P1 and P2, whilst the relative intensity is less informative. As shown in Figure S22, the phase shift observed at 160 K is a clear indication of the validity of the cold-finger cryostat measurement and P1 alone suffices to determine the temperature of the $MAPbI_3$ single crystal accurately.

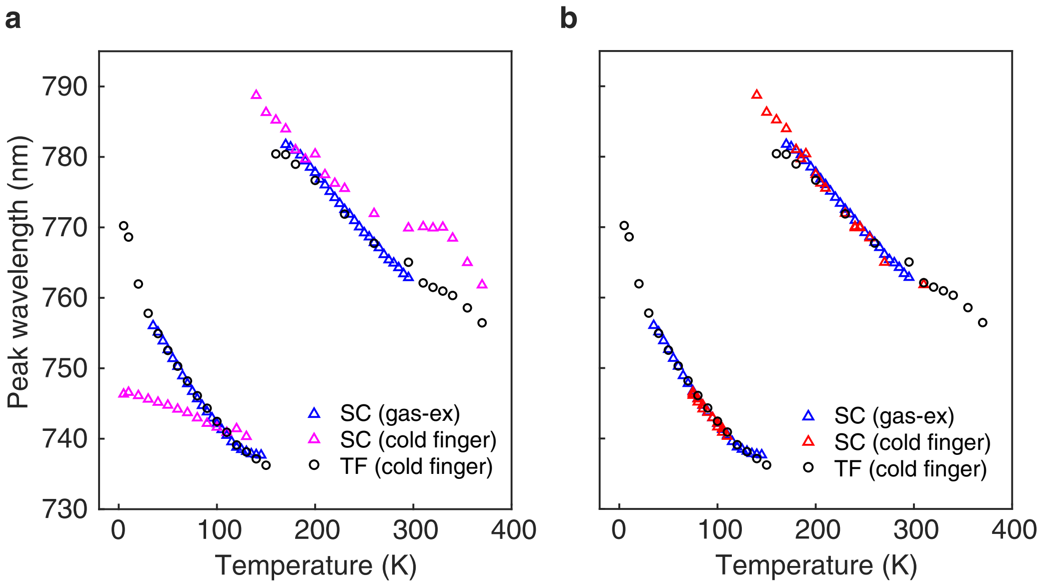

Figure S22: (a) Central wavelength of P1 before temperature correction. (b) Central wavelength of P1 after temperature correction. The blue triangles represent the P1 positions of the single crystal measured by the gas-exchange cryostat, which is used as the true temperature reference. The black circles represent the P1 positions of the thin film measured by the cold-finger cryostat, which shows a good agreement with the single crystal's gas-exchange measurement. Hence, there is no need to correct the temperature of the thin film measurements. The pink and red triangles represent the cold-finger cryostat measurements of the single crystal before and after temperature correction respectively.

References

(S1) Seifert, T.; Jaiswal, S.; Sajadi, M.; Jakob, G.; Winnerl, S.; Wolf, M.; Kläui, M.; Kampfrath, T. Ultrabroadband Single-Cycle Terahertz Pulses with Peak Fields of 300 kVcm^{-1} from A Metallic Spintronic Emitter. *Appl. Phys. Lett.* **2017**, *110*, 252402.

(S2) Herz, L. M. How Lattice Dynamics Moderate the Electronic Properties of Metal-Halide Perovskites. *J. Phys. Chem. Lett.* **2018**, *9*, 6853–6863.

(S3) Poglitsch, A.; Weber, D. Dynamic Disorder in Methylammoniumtrihalogenoplumbates (II) Observed by Millimeter-Wave Spectroscopy. *J. Chem. Phys.* **1987**, *87*, 6373–6378.

(S4) Sendner, M.; Nayak, P. K.; Egger, D. A.; Beck, S.; Müller, C.; Epding, B.; Kowalsky, W.; Kronik, L.; Snaith, H. J.; Pucci, A., et al. Optical Phonons in Methylammonium Lead Halide Perovskites and Implications for Charge Transport. *Mater. Horiz.* **2016**, *3*, 613–620.

(S5) Ulatowski, A. M.; Herz, L. M.; Johnston, M. B. Terahertz Conductivity Analysis for Highly Doped Thin-Film Semiconductors. *J. Infrared Millim. Terahertz Waves* **2020**, *41*, 1431–1449.

(S6) Kužel, P.; Němec, H. Terahertz Conductivity in Nanoscaled Systems: Effective Medium Theory Aspects. *J. Phys. D Appl. Phys.* **2014**, *47*, 374005.

(S7) Hempel, H.; Unold, T.; Eichberger, R. Measurement of Charge Carrier Mobilities in Thin Films on Metal Substrates by Reflection Time Resolved Terahertz Spectroscopy. *Opt. Express* **2017**, *25*, 17227–17236.

(S8) Wehrenfennig, C.; Liu, M.; Snaith, H. J.; Johnston, M. B.; Herz, L. M. Charge-Carrier Dynamics in Vapour-Deposited Films of the Organolead Halide Perovskite $CH_3NH_3PbI_{3-x}Cl_x$. *Energy Environ. Sci.* **2014**, *7*, 2269–2275.

(S9) Wehrenfennig, C.; Eperon, G. E.; Johnston, M. B.; Snaith, H. J.; Herz, L. M. High Charge Carrier Mobilities and Lifetimes in Organolead Trihalide Perovskites. *Adv. Mater.* **2014**, *26*, 1584–1589.

(S10) Milot, R. L.; Eperon, G. E.; Snaith, H. J.; Johnston, M. B.; Herz, L. M. Temperature-Dependent Charge-Carrier Dynamics in $CH_3NH_3PbI_3$ Perovskite Thin Films. *Adv. Funct. Mater.* **2015**, *25*, 6218–6227.

(S11) D'Angelo, F.; Němec, H.; Parekh, S. H.; Kužel, P.; Bonn, M.; Turchinovich, D. Self-Referenced Ultra-Broadband Transient Terahertz Spectroscopy Using Air-Photonics. *Opt. Express* **2016**, *24*, 10157–10171.

(S12) Leguy, A. M.; Hu, Y.; Campoy-Quiles, M.; Alonso, M. I.; Weber, O. J.; Azarhoosh, P.; Van Schilfgaarde, M.; Weller, M. T.; Bein, T.; Nelson, J., et al. Reversible Hydration of $CH_3NH_3PbI_3$ in Films, Single Crystals, and Solar Cells. *Chem. Mater.* **2015**, *27*, 3397–3407.

(S13) Stoumpos, C. C.; Malliakas, C. D.; Kanatzidis, M. G. Semiconducting Tin and Lead Iodide Perovskites with Organic Cations: Phase Transitions, High Mobilities, and Near-Infrared Photoluminescent Properties. *Inorg. Chem.* **2013**, *52*, 9019–9038.

(S14) Dong, Q.; Fang, Y.; Shao, Y.; Mulligan, P.; Qiu, J.; Cao, L.; Huang, J. Electron-Hole Diffusion Lengths $> 175 \mu m$ in Solution-Grown $CH_3NH_3PbI_3$ Single Crystals. *Science* **2015**, *347*, 967–970.

(S15) Shi, D.; Adinolfi, V.; Comin, R.; Yuan, M.; Alarousu, E.; Buin, A.; Chen, Y.; Hoogland, S.; Rothenberger, A.; Katsiev, K., et al. Low Trap-State Density and Long Carrier Diffusion in Organolead Trihalide Perovskite Single Crystals. *Science* **2015**, *347*, 519–522.

(S16) Saidaminov, M. I.; Abdelhady, A. L.; Murali, B.; Alarousu, E.; Burlakov, V. M.; Peng, W.; Dursun, I.; Wang, L.; He, Y.; Maculan, G., et al. High-Quality Bulk Hybrid

Perovskite Single Crystals within Minutes by Inverse Temperature Crystallization. *Nat. Commun.* **2015**, *6*, 1–6.

(S17) Shrestha, S.; Matt, G. J.; Osvet, A.; Niesner, D.; Hock, R.; Brabec, C. J. Assessing temperature dependence of drift mobility in methylammonium lead iodide perovskite single crystals. *The Journal of Physical Chemistry C* **2018**, *122*, 5935–5939.

(S18) Senanayak, S. P.; Yang, B.; Thomas, T. H.; Giesbrecht, N.; Huang, W.; Gann, E.; Nair, B.; Goedel, K.; Guha, S.; Moya, X., et al. Understanding Charge Transport in Lead Iodide Perovskite Thin-Film Field-Effect Transistors. *Sci. Adv.* **2017**, *3*, e1601935.

(S19) Yu, W.; Li, F.; Yu, L.; Niazi, M. R.; Zou, Y.; Corzo, D.; Basu, A.; Ma, C.; Dey, S.; Tietze, M. L., et al. Single Crystal Hybrid Perovskite Field-Effect Transistors. *Nat. Commun.* **2018**, *9*, 1–10.

(S20) Semonin, O. E.; Elbaz, G. A.; Straus, D. B.; Hull, T. D.; Paley, D. W.; Van der Zande, A. M.; Hone, J. C.; Kymissis, I.; Kagan, C. R.; Roy, X., et al. Limits of Carrier Diffusion in n-Type and p-Type $CH_3NH_3PbI_3$ Perovskite Single Crystals. *J. Phys. Chem. Lett.* **2016**, *7*, 3510–3518.

(S21) Hutter, E. M.; Eperon, G. E.; Stranks, S. D.; Savenije, T. J. Charge Carriers in Planar and Meso-Structured Organic-Inorganic Perovskites: Mobilities, Lifetimes, and Concentrations of Trap States. *J. Phys. Chem. Lett.* **2015**, *6*, 3082–3090.

(S22) Reid, O. G.; Yang, M.; Kopidakis, N.; Zhu, K.; Rumbles, G. Grain-Size-Limited Mobility in Methylammonium Lead Iodide Perovskite Thin Films. *ACS Energy Lett.* **2016**, *1*, 561–565.

(S23) Valverde-Chávez, D. A.; Ponseca, C. S.; Stoumpos, C. C.; Yartsev, A.; Kanatzidis, M. G.; Sundström, V.; Cooke, D. G. Intrinsic Femtosecond Charge Gen-

eration Dynamics in Single Crystal $CH_3NH_3PbI_3$. *Energy Environ. Sci.* **2015**, *8*, 3700–3707.

(S24) Karakus, M.; Jensen, S. A.; D'Angelo, F.; Turchinovich, D.; Bonn, M.; Canovas, E. Phonon-Electron Scattering Limits Free Charge Mobility in Methylammonium Lead Iodide Perovskites. *J. Phys. Chem. Lett.* **2015**, *6*, 4991–4996.

(S25) Poncé, S.; Schlipf, M.; Giustino, F. Origin of Low Carrier Mobilities in Halide Perovskites. *ACS Energy Lett.* **2019**, *4*, 456–463.

(S26) Steinhögl, W.; Schindler, G.; Steinlesberger, G.; Engelhardt, M. Size-Dependent Resistivity of Metallic Wires in the Mesoscopic Range. *Phys. Rev. B* **2002**, *66*, 075414.

(S27) Sun, T.; Yao, B.; Warren, A. P.; Barmak, K.; Toney, M. F.; Peale, R. E.; Coffey, K. R. Surface and Grain-Boundary Scattering in Nanometric Cu Films. *Phys. Rev. B* **2010**, *81*, 155454.

(S28) Schneider, C. A.; Rasband, W. S.; Eliceiri, K. W. NIH Image to ImageJ: 25 Years of Image Analysis. *Nat. Methods* **2012**, *9*, 671–675.

(S29) Jariwala, S.; Sun, H.; Adhyaksa, G. W.; Lof, A.; Muscarella, L. A.; Ehrler, B.; Garnett, E. C.; Ginger, D. S. Local Crystal Misorientation Influences Non-Radiative Recombination in Halide Perovskites. *Joule* **2019**, *3*, 3048–3060.

(S30) Patterson, A. The Scherrer Formula for X-Ray Particle Size Determination. *Phys. Rev.* **1939**, *56*, 978.

(S31) Crothers, T. W.; Milot, R. L.; Patel, J. B.; Parrott, E. S.; Schlipf, J.; Müller-Buschbaum, P.; Johnston, M. B.; Herz, L. M. Photon Reabsorption Masks Intrinsic Bimolecular Charge-Carrier Recombination in $CH_3NH_3PbI_3$ Perovskite. *Nano Lett.* **2017**, *17*, 5782–5789.

(S32) Johnston, M. B.; Herz, L. M. Hybrid Perovskites for Photovoltaics: Charge-Carrier Recombination, Diffusion, and Radiative Efficiencies. *Acc. Chem. Res.* **2016**, *49*, 146–154.
